# BAP1 is a haploinsufficient tumor suppressor linking chronic pancreatitis to pancreatic cancer in mice

**DOI:** 10.1038/s41467-020-16589-8

**Published:** 2020-06-15

**Authors:** Stephanie Perkail, Jaclyn Andricovich, Yan Kai, Alexandros Tzatsos

**Affiliations:** 1 Cancer Epigenetics Laboratory, Department of Anatomy and Cell Biology, GWU School of Medicine and Health Sciences, Washington, DC 20052 USA; 2 George Washington University (GWU) Cancer Center, GWU School of Medicine and Health Sciences, Washington, DC 20052 USA; 3 Present Address: Department of Biostatistics and Computational Biology, Dana-Farber Cancer Institute, Boston, MA USA

**Keywords:** Cancer, Genetics

## Abstract

Chronic pancreatitis represents a risk factor for the development of pancreatic cancer. We find that heterozygous loss of histone H2A lysine 119 deubiquitinase BAP1 (BRCA1 Associated Protein-1) associates with a history of chronic pancreatitis and occurs in 25% of pancreatic ductal adenocarcinomas and 40% of acinar cell carcinomas. Deletion or heterozygous loss of *Bap1* in murine pancreata causes genomic instability, tissue damage, and pancreatitis with full penetrance. Concomitant expression of *Kras*^*G12D*^ leads to predominantly intraductal papillary mucinous neoplasms and mucinous cystic neoplasms, while pancreatic intraepithelial neoplasias are rarely detected. These lesions progress to metastatic pancreatic cancer with high frequency. Lesions with histological features mimicking Acinar Cell Carcinomas are also observed in some tumors. Heterozygous mice also develop pancreatic cancer suggesting a haploinsufficient tumor suppressor role for BAP1. Mechanistically, BAP1 regulates genomic stability, in a catalytic independent manner, and its loss confers sensitivity to irradiation and platinum-based chemotherapy in pancreatic cancer.

## Abstract (web summary)

Chronic pancreatitis is linked with an increased risk of pancreatic cancer. Here the authors show that genomic instability due to the loss of BRCA1 associated protein-1, frequently observed in patients with pancreatic cancer, is associated with chronic pancreatitis and predisposes to cancer development.

## Introduction

Pancreatic ductal adenocarcinoma (PDA) is a lethal malignancy with a 5-year survival rate of 10%. Sequencing of pancreatic cancer genomes revealed clinically relevant molecular subtypes^1–3^. Based on structural variations at the chromosomal level, PDA has been classified into four subtypes: stable, locally rearranged, scattered, and unstable^4^. The latter is driven by mutations in DNA-damage repair genes, such as *BRCA1*, *BRCA2*, *ATM*, and *PALB2*, and responds to treatments triggering DNA damage, such platinum-based chemotherapy, poly (ADP-ribose) polymerase (PARP) inhibitors, and irradiation (IR)^4–8^. Consistently, targeted inactivation of either *Brca2* or *Atm* in murine pancreata caused genomic instability and promoted aggressive cancer that was sensitive to IR^7–10^. In early stages of PDA, the activation of DNA damage response (DDR) triggered by oncogenic Kras poses a barrier to cancer development. However, progressive failure of DNA repair pathways can facilitate malignant transformation and tumor evolution. Thus, further identification of molecular defects compromising genome integrity may indicate novel biomarkers of responsiveness to systemic chemotherapy and refine rational approaches for therapeutic intervention.

FOLFIRINOX (5-Fluorouracil, Leucovorin, Irinotecan, and Oxaliplatin), a platinum- and topoisomerase I inhibitor-containing combination therapy is emerging as a first-line treatment for patients with good performance status, who are diagnosed with PDA^11^. Intriguingly, although improved survival has been reported for patients with mutations in DNA repair genes^4,6^, the efficacy of FOLFIRINOX extends beyond the small percentage of cases exhibiting mutations of *BRCA1/2*, *PALB2*, and *ATM*, indicating that defects in other genes regulating the DDR may contribute as well. This observation motivated us to analyze pancreatic cancer genomes for copy number alterations (CNAs) of genes implicated in DNA repair. We found that heterozygous loss of *BAP1*—but rarely mutation—occurs in over a quarter of PDAs and 40% of acinar cell carcinomas (ACCs), a rare and distinct subtype of pancreatic cancer that infrequently harbors *KRAS* mutations. *BAP1* is located on the short arm of chromosome 3 (3p21.1) where loss of heterozygosity (LOH) or deletion is a recurrent and early event in the formation of multiple tumors^12^. Furthermore, *BAP1* germline mutations cause a tumor predisposition syndrome inherited in an autosomal dominant pattern, which confers a higher risk for developing a spectrum of benign and malignant tumors^13,14^, including pancreatic cancer^13,15–18^.

BAP1 interacts with ASXL proteins to form the polycomb repressive deubiquitinase (PR-DUB) complex, which counteracts the function of polycomb repressor complex 1 (PRC1) through histone H2AK119 deubiquitination to regulate developmental pathways^19^. BAP1 also participates in multiprotein complexes to regulate gene expression and cell proliferation^20^. Intriguingly, BAP1 is directly phosphorylated by ATM upon DNA damage and is required for efficient assembly of homologous recombination (HR) repair factors BRCA1 and RAD51 at IR-induced foci to promote repair of DNA double-strand breaks (DSBs)^21,22^. Although heterozygous loss of *BAP1* is frequent in pancreatic cancer, the molecular mechanisms underlying this aberration in pancreas homeostasis and tumorigenesis remain unknown. Here we find that genomic instability due to the loss of *BAP1* is frequently observed in patients with pancreatic cancer and is associated with a history of chronic pancreatitis.

## Results

### BAP1 loss associates with pancreatitis and a poor prognosis

**Fig. 1:**
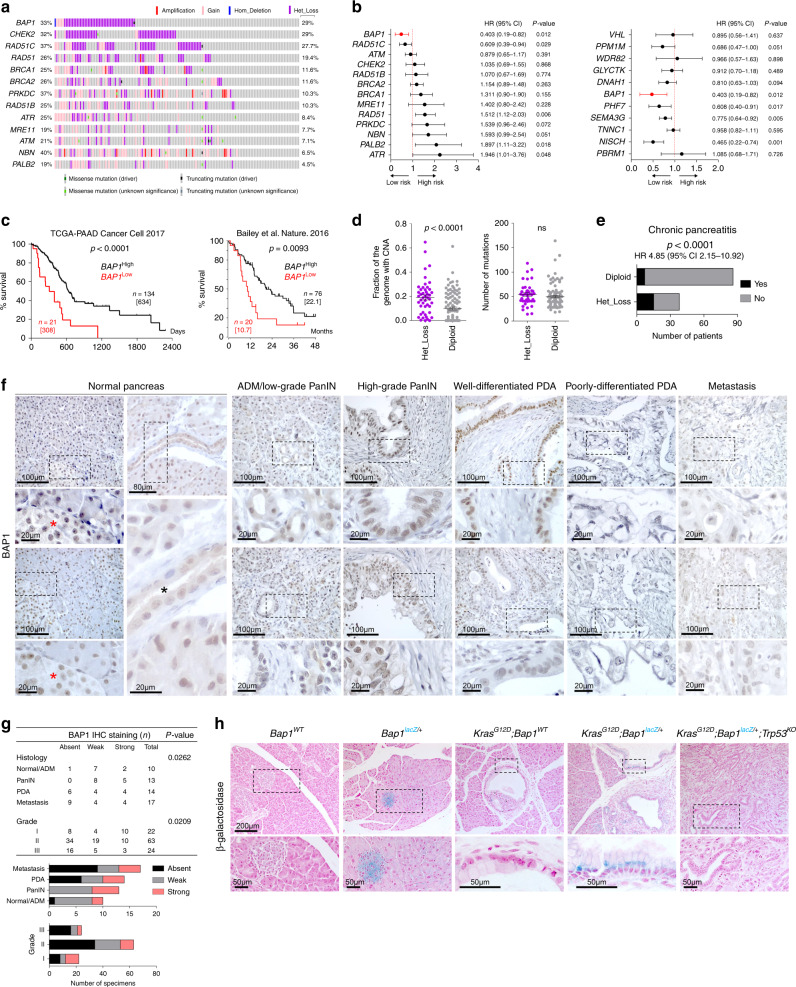
BAP1 loss confers a dismal prognosis in PDA. **a** Oncoprint showing the percentage of patients with copy number alterations (left) and heterozygous loss (right) for the indicated genes in the TCGA-PAAD cohort. **b** Forest plot showing the log hazard ratio (HR) and 95% confidence intervals (CIs) for death in the TCGA-PAAD cohort (*n* = 155 patients) estimated by univariate Cox regression analysis for the expression of genes involved in DNA repair (left) and located adjacent to *BAP1* (right). The *p*-values were calculated based on the *z*-scores determined by Wald’s test. **c** Kaplan–Meier plots showing the survival of patients in the indicated studies stratified based on *BAP1* expression. Median survival is shown in brackets. *n*, number of patients. The Log-rank (Mantel–Cox) test was used to assess statistical significance between groups. **d** Scatter dot plots (mean ± SEM) showing the fraction of genome carrying copy number alterations (CNA, left) and mutations (right) in TCGA-PAAD patients with heterozygous loss of *BAP1*. Statistical significance was determined by a two-tailed unpaired Student’s *t*-test. ns, nonsignificant. **e** Stacked bar graph showing the number of patients in the TCGA-PAAD cohort with evidence of chronic pancreatitis and heterozygous loss of *BAP1*. Statistical significance was determined by a two-sided Fisher’s exact test. (“Yes;HET_loss” *n* = 15, “NO;HET_loss” *n* = 23, “Yes;Diploid” *n* = 7, “NO;Diploid” *n* = 79). **f** Representative IHC of BAP1 in human pancreatic cancer tissue microarrays representing normal pancreas, early and advanced premalignant lesions, well- and poorly differentiated pancreatic ductal adenocarcinoma (PDA), and metastatic disease in the liver. ADM, acinar-to-ductal metaplasia; PanIN, pancreatic intraepithelial neoplasia. Red and black asterisks indicate islets and a duct, respectively. **g** Contingency table (top) and stacked bar graphs (bottom) showing the staining intensity of BAP1 with regard to histology and tumor grade. Statistical significance was determined by a *χ*^2^-test. **h** β-Galactosidase staining in pancreata of the indicated genotypes at 8–10 weeks of age. Source data are provided as a Source Data file.

Interrogation of the TCGA-PAAD cohort^2^ for mutations and CNAs revealed frequent heterozygous loss for genes implicated in DDR, particularly for *BAP1* (29%), *CHEK2* (29%), and *RAD51C* (27.7%) (Fig. 1a). Notably, genomic losses of *BAP1* were mainly present in high-purity samples (*p* = 0.0178; two-sided Fisher’s exact test), indicating that the frequency and depth of deletion may be underestimated in the TCGA-PAAD cohort (Supplementary Fig. 1a). Several patients simultaneously carried multiple hits, indicating that defective DNA repair is a common molecular denominator in PDA. Although heterozygous loss was accompanied by reduced transcript levels for several genes involved in DDR (Supplementary Fig. 1b), only *BAP1* and *RAD51C* expression were significantly associated with lower risk and longer survival (Fig. 1b). Surprisingly, the expression of other DNA repair genes did not significantly correlate with survival (*ATM* and *BRCA1/2*) or had an adverse outcome (*RAD51*, *PALB2*, and *ATR*). The expression of *PHF7*, *SEMA3G*, and *NISCH*, which are adjacent to *BAP1* and affected by LOH of Chr 3p21.1, were also associated with lower risk and longer survival in the TCGA-PAAD cohort (Fig. 1b). On the other hand, no correlation was found with the expression of *PBRM1*, a well-known tumor suppressor located in the same cytogenetic band as *BAP1*, as well as *VHL*, which is also located on the short arm of chromosome 3 (Chr 3p25.3). Although *BAP1* expression is not linked to a pancreatic cancer subtype^1^ (Supplementary Fig. 1c), in two independent pancreatic cancer cohorts^1,2^ patients with low expression of *BAP1* exhibited shorter median survival and a dismal prognosis (hazard ratio (HR) 4.94 with 95% confidence interval (95% CI) 2.62–10.79, Fig. 1c). Meta-analysis of additional studies confirmed that heterozygous loss, but rarely mutations or deletion, of *BAP1* is a common aberration in >25% of PDA patients^2,3,23^ (Supplementary Fig. 1d). Heterozygous loss of *BAP1* was also present in (a) 40% of ACC, which also exhibit copy number losses for several DNA repair genes^23,24^, and (b) 15–20% of pancreatic tumors arising from mucinous cystic neoplasms (MCNs), intraductal papillary mucinous neoplasms (IPMNs), and solid pseudopapillary neoplasms^23,25,26^ (Supplementary Fig. 1d, e). Consistently, most human PDA cell lines show heterozygous loss of *BAP1* and express reduced mRNA and protein compared with cell lines established from other malignancies (Supplementary Fig. 1f, g). Patients with loss of *BAP1* exhibited more than a twofold increase in the fraction of the genome carrying CNAs, but no difference in the mutational burden (Fig. 1d), and were associated with a history of chronic pancreatitis (Fig. 1e). The latter group also showed enrichment for CNAs but not mutations (Supplementary Fig. 1h). Thus, genomic instability due to BAP1 deficiency may be associated to the development of chronic pancreatitis and pancreatic cancer.

Next, we stained human PDA tissue microarrays (TMAs) comprising specimens representing progression of premalignant lesions to overt cancer and metastatic disease, and stratified based on grade (well- vs. poorly differentiated, Fig. 1f), with a BAP1 antibody that was validated for immunohistochemistry (IHC) in knockout mice (Supplementary Fig. 1i). In human and mouse normal pancreata, BAP1 is expressed in islets and displays a mosaic pattern in acinar cells (Fig. 1f and Supplementary Fig. 1i, j). BAP1 is expressed in acinar-to-ductal metaplasia and premalignant pancreatic intraepithelial neoplasias (PanINs), as well as in some cases of well-differentiated PDA. In contrast, BAP1 was absent, or downregulated, in the majority of poorly differentiated (grade III) specimens, whereas a statistically significant inverse correlation was observed between BAP1 staining and tumor grade, as well as progression to metastatic disease (Fig. 1f–g).

To study the expression of endogenous *Bap1* in a progression model of pancreatic cancer, we established a knockin reporter mouse carrying a *lacZ* cassette under the control of the endogenous *Bap1* promoter flanked by loxP sites to allow tissue-specific deletion following intercrossing with the *Pdx1*^*Cre*^ strain^27^ (Supplementary Fig. 1k). Early in life, *Bap1*^*lacZ/+*^ mice exhibited normal endocrine and exocrine pancreas histology and showed widespread expression of β-galactosidase (Supplementary Fig. 1l). On the contrary, β-galactosidase in older mice was largely confined to islets with a weak mosaic staining of acinar and ductal cells. Consistent with the expression pattern observed in human (Fig. 1f), β-galactosidase staining in murine pancreata showed an increase in premalignant lesions driven by *Kras*^*G12D*^, but no staining was detected in overt cancer upon concomitant loss of *Trp53* (Fig. 1h and IHC in Supplementary Fig. 1m).

### Loss of Bap1 induces DNA damage and chronic pancreatitis

**Fig. 2:**
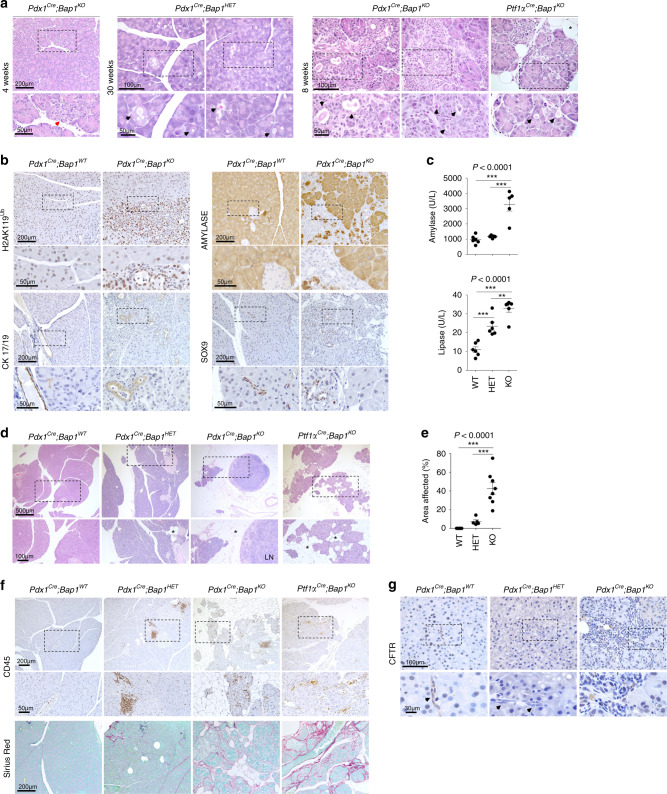
BAP1 loss induces pancreatitis. **a** H&E staining of pancreata from the indicated genotypes and ages. Red arrow points to an area with loss of acinar architecture and black arrows the accumulation of eosinophilic zymogen material and luminal dilation of acini. **b** IHC for the indicated markers in wild-type and *Bap1*-knockout mice at 8–10 weeks of age. The *Pdx1*^*Cre*^ strain expresses *Cre* recombinase in a stochastic pattern causing mosaic histological alterations due to loss of *Bap1*. **c** Scatter dot plots (mean ± SEM) show serum amylase and lipase levels in 25–30-week-old mice of the indicated genotypes. (Amylase: WT *n* = 6, HET *n* = 5, and KO *n* = 5 mice. Lipase: WT *n* = 6, HET *n* = 6, and KO *n* = 6 mice). **d** H&E staining of 30-week-old pancreata of the indicated genotypes. Asterisks indicate saponification. LN, lymph node. **e** Scatter dot plot (mean ± SEM) showing the percentage of affected area in 30-week-old pancreata of the indicated genotypes (WT *n* = 6, HET = 5, KO *n* = 8 mice). **f** IHC for CD45 (top) and Sirius Red staining (bottom) showing leukocyte infiltration and collagen deposition, respectively, into the pancreatic parenchyma of 30-week-old wild-type and *Bap1*-deficient mice. **g** IHC for CFTR in 8–10-week-old wild-type and *Bap1*-deficient mice. Arrows point to ducts. In **c** and **e**, statistical significance was determined by one-way ANOVA with *p*-values shown on the top of each plot, followed by Tukey’s multiple comparison post-hoc test between groups test. **p < 0.01; ***p < 0.001. Source data are provided as a Source Data file.

We generated pancreas-specific conditional knockout mice by sequentially crossing *Bap1*^*lacZ/+*^ with *β-Actin*^*FLPe*^ animals to remove the *Frt* cassette and crossed the offspring to *Pdx1*^*Cre*^^28^ or *Ptf1α*^*Cre*^^29^ mice to delete *Bap1* in pancreas progenitor cells (Supplementary Fig. 2a). Pancreas-specific *Bap1*-null mice were born at the expected gender and genotype ratios. Genotyping confirmed recombination in genomic DNA isolated from the pancreas, but not the tail, and IHC confirmed the mosaic absence of Bap1 in the exocrine pancreas (Supplementary Fig. 2b and 1i). Islets retained expression of Bap1 and mice exhibited normal glucose homeostasis (Supplementary Fig. 2c, d). Histological analyses of knockout pancreata at 4 weeks of age revealed focal areas with loss of acinar architecture that over time evolved to variable hyperplastic, metaplastic, and connective tissue changes accompanied by obstruction of the ducts with eosinophilic zymogen material, which caused luminal dilation of acini and reactive proliferation (Fig. 2a and Supplementary Fig. 2e). Similar changes were also observed in heterozygous (Fig. 2a) and *Bap1*^*lacZ/+*^ pancreata (Supplementary Fig. 2f). IHC revealed a strong upregulation of H2A ubiquitinated at Lys119 (H2AK119Ub), loss of acinar identity (Amylase), and expansion of Cytokeratin 17/19- and Sox9-positive duct-like cells (Fig. 2b). After 20 weeks of age, knockout out mice presented with catatonic behavior, hunched posture, and poor grooming, rendering them moribund. Post-mortem examination revealed elevated levels of serum amylase and lipase (Fig. 2c), a reduction in pancreas size with loss of tissue integrity and a gelatinous appearance (Supplementary Fig. 2g). Microscopic findings included loss of acini, immune cell infiltration, fibrosis (Sirius Red staining), and saponification, indicative of tissue inflammation and damage (Fig. 2d–f). Consistently, IHC and flow cytometry confirmed infiltration by T cells (CD3^+^), dendritic cells (CD11c^+^), and macrophages (F4/80^+^ and CD11b^+^), but not B cells (B220^+^) (Supplementary Fig. 2h, i). The histologic changes—accumulation of insoluble eosinophilic material and inflammation—were reminiscent of chronic pancreatitis secondary to cystic fibrosis, a genetic disorder caused by mutations in the cystic fibrosis transmembrane conductance regulator (*CFTR*) gene. Cystic fibrosis patients exhibit higher risk for chronic pancreatitis and PDA^30,31^. *Cftr*, which is primarily expressed in the duct epithelia and a very small percentage of acinar cells (Supplementary Fig. 2j), was downregulated in *Bap1* heterozygotes and was absent in the expanded duct-like cells of *Bap1*-null pancreata (Fig. 2g).

**Fig. 3:**
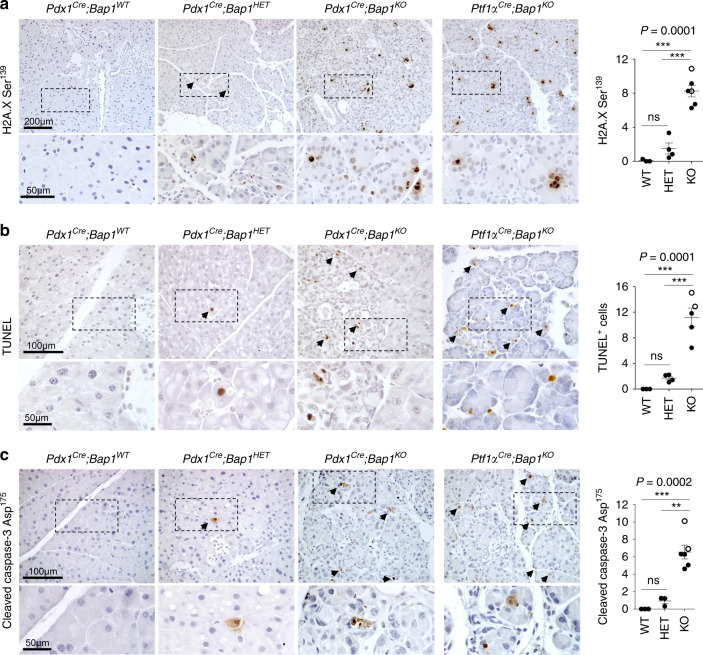
BAP1 loss triggers a DNA damage response. **a** H2A.X phosphorylated at Ser139 (WT *n* = 3, HET *n* = 4, KO *n* = 6 mice), **b** TUNEL (WT *n* = 3, HET *n* = 4, KO *n* = 5 mice), and **c** cleaved Caspase-3 IHC (WT *n* = 3, HET *n* = 3, KO *n* = 6 mice) in 8–10-week-old mice of the indicated genotypes. Arrows indicate positively stained cells. Right: scatter dot plots showing the number of positive cells (mean ± SEM) per 0.1 mm^2^ of tissue per mouse. Each dot represents a mouse. Filled and open circles indicate mice from the *Pdx1*^*Cre*^ and *Ptf1α*^*Cre*^ cohorts, respectively. Mice lacking the *Cre* allele were used as control for both cohorts. Statistical significance was determined by one-way ANOVA with *p*-values shown on the top of each plot, followed by Tukey’s multiple comparison post-hoc test between groups. ns, nonsignificant; **p < 0.01; ***p < 0.001. Source data are provided as a Source Data file.

We reasoned that BAP1 ablation might trigger chronic tissue damage and pancreatitis due to defective DDR. Indeed, we found a strong increase in the number of cells positive for H2A.X phosphorylated at Ser139 (H2A.XSer139, Fig. 3a), which occurs in response to DNA damage by ATM, ATR, or DNA-PK kinases and signals the recruitment of DNA-repair proteins. We also detected an increase of cells with apoptotic DNA fragmentation (terminal deoxynucleotidyl transferase‐mediated dUTP nick‐end labeling (TUNEL), Fig. 3b) and cleaved Caspase-3 (Fig. 3c). At the molecular level, meta-analysis of gene expression profiles of acute pancreatitis in caerulein-treated mice^32,33^ revealed that *Bap1* and other DNA repair genes (*Brca1/2* and *Atm*) are upregulated within hours in response to tissue damage (Supplementary Fig. 3a, b). Transcriptional activation of the endogenous *Bap1* locus was also confirmed in caerulein-treated *Bap1*^*lacZ/+*^ mice (Supplementary Fig. 3c), suggesting that DDR is part of the physiological response for tissue regeneration. Consistently, caerulein-treated *Bap1* heterozygous and knockout mice showed defective tissue regeneration, persistent activation of DDR, immune cell infiltration, and ductal metaplasia (Supplementary Fig. 3d–f).

### BAP1 restrains *Kras*^*G12D*^-driven pancreatic cancer

**Fig. 4:**
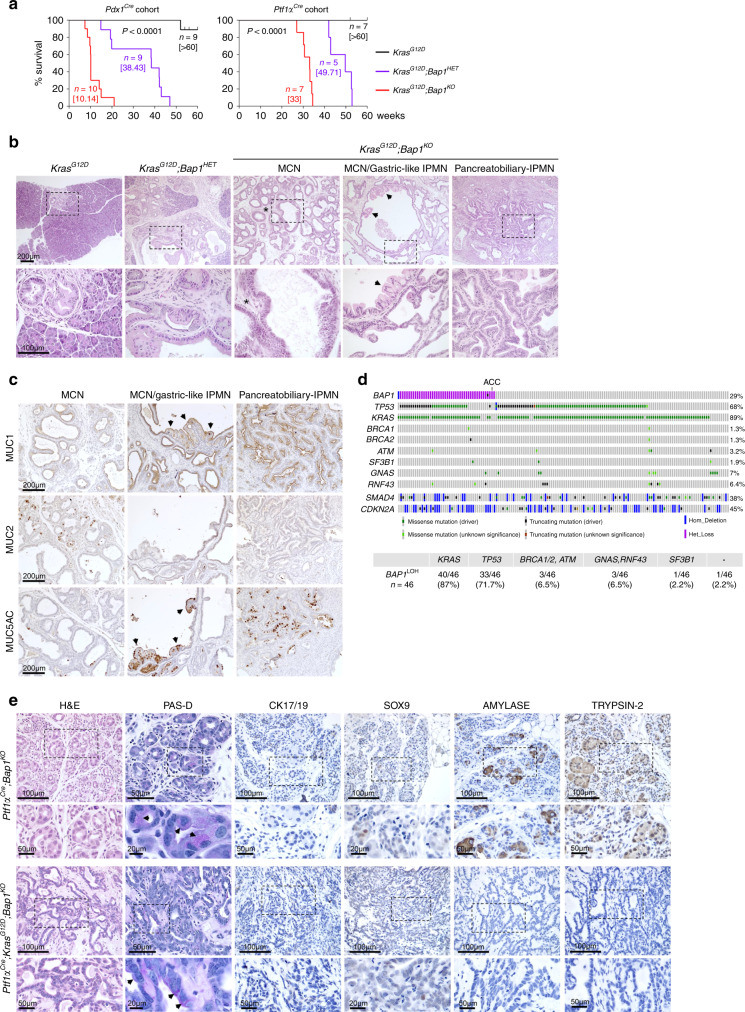
BAP1 restrains KRAS^G12D^ driven pancreatic cancer. **a** Kaplan–Meier plots showing the survival of mice of the indicated genotypes in the *Pdx1*^*Cre*^*;Kras*^*G12D*^ (left) and *Ptf1α*^*Cre*^*;Kras*^*G12D*^ (right) cohorts. Median survival is shown in brackets. The Log-rank (Mantel–Cox) test was used to assess statistical significance between groups. *n*, number of mice. **b** H&E staining of 10–15-week-old pancreata from the *Pdx1*^*Cre*^ cohort lacking *Bap1* with histological alterations resembling mucinous cystic neoplasms (MCNs) and intraductal papillary mucinous neoplasms (IPMNs). Asterisks indicate ovarian type stroma lined by mucin-producing cells in MCN and arrows point to the foveolar epithelium. **c** IHC for the indicated mucins in *Pdx1*^*Cre*^*;Kras*^*G12D*^*;Bap1*^*KO*^ tumors isolated from 10- to 15-week-old mice. Arrows point to areas of the foveolar epithelium. **d** Top: Oncoprint showing the overlap between heterozygous loss of BAP1 and mutations in the indicated oncogenes and tumor suppressors in the TCGA-PAAD cohort. Bottom: Table showing the percentage of patients with heterozygous loss of *BAP1* and mutations in the indicated genes. ACC, acinar cell carcinoma. **e** H&E, Periodic Acid–Schiff staining with Diastase digestion (PAS-D) and IHC for ductal (Cytokeratin 17/19 and Sox9) and acinar (Amylase and Trypsin-2) markers in 25–30-week-old mice of the indicated genotypes. Arrows point to PAS-D-positive cytoplasmic staining of cells in areas with histological features resembling ACC. Source data are provided as a Source Data file.

Targeted activation of *Kras*^*G12D*^ in murine pancreas induces PanINs, which progress to cancer with a long latency^28^. In contrast, caerulein-induced pancreatitis accelerates *Kras*^*G12D*^-driven PanIN progression to cancer as early as 1 month post treatment, suggesting that tissue inflammation establishes a pro-oncogenic milieu^34^. We found that deletion and heterozygous loss of *Bap1* in *Pdx1*^*Cre*^*;Kras*^*G12D*^ and *Ptf1α*^*Cre*^*;Kras*^*G12D*^ mice accelerated cancer progression and shortened survival (Fig. 4a). Macroscopic inspection of knockout pancreata revealed cystic lesions of variable size in all mice (Supplementary Fig. 4a). Histology resembled MCNs with the presence of large multilocular cysts surrounded by ovarian type stroma (Fig. 4b). In several mice, we also observed a spectrum of IPMN-like lesions or mixed MCN-IPMN histology with an intraductal proliferation of mucin-producing cells, the formation of papillae, and cystic dilatation of the pancreatic ducts (Fig. 4b). On the other hand, heterozygous tumors showed features of both PDA and cystic alterations. Interestingly, although they retained the wild-type allele, IHC showed mosaic staining and downregulation of Bap1 in cystic lesions (Supplementary Fig. 4b). Thus, Bap1 restrains *Kras*^*G12D*^-driven cell transformation and results in a higher frequency of IPMN and MCN histology. *Kras*^*G12D*^*;Bap1*-knockout pancreata exhibited increases in histone H2AK119Ub, loss of acini (Amylase), and gain of duct-like fate (Cytokeratin 17/19 and Sox9) (Supplementary Fig. 4c, d). MCNs were variably positive for Muc1 and Muc5AC, which correlate with a poor prognosis in humans^35^, and less frequently for Muc2, a marker of goblet cell metaplasia (Fig. 4c). IPMNs were more frequently positive and stained stronger for Muc1 and Muc5AC, and exhibited marked cytological atypia and had a complex cribriform architecture similar to gastric-like and pancreatobiliary IPMNs (Fig. 4b, c). Serial sectioning and histological analyses of all knockout animals revealed tumor invasion of pancreatic lymph nodes and micro-metastases in the liver (Supplementary Fig. 4e). Although with slower kinetics, all heterozygous mice also presented with lymph node invasion and distal metastases in the liver. Lung metastases were also observed in two mice (Supplementary Fig. 4f).

To confirm these findings, we examined the tumor histology and pathology reports through the Cancer Digital Slide Archive (http://cancer.digitalslidearchive.net) and found that although the majority of *KRAS* mutant TCGA-PAAD patients with heterozygous loss of *BAP1* harbored mutations of *TP53* and developed PDA, 6/7 patients with wild-type *TP53* presented with pancreatic cancer arising from IPMNs and MCNs (Fig. 4d and Supplementary Fig. 4g). Patients with heterozygous loss of *BAP1* had sporadic mutations of unknown significance in *BRCA1/2*, *ATM*, and *SF3B1*—which are very rare in the TCGA-PAAD cohort, and were mutual exclusive with mutations of *GNAS*, which is a known driver of cystic pancreatic neoplasms^36,37^ (Supplementary Fig. 4h). All patients with wild-type *KRAS* and *TP53* that harbor heterozygous loss of *BAP1* presented with IPMNs and MCNs (Supplementary Fig. 4i). Last, the only patient carrying a truncating mutation of BAP1 and oncogenic *KRAS* presented with extensive parenchyma inflammation and metaplastic alterations (Supplementary Fig. 4j).

Exclusively in *Ptf1α*^*Cre*^*;Bap1*^*KO*^ mice, which recombine predominantly in the acinar compartment and to a lesser extent in ductal cells, we observed structures resembling normal acini with cells containing large nuclei, prominent nucleoli, and a finely granular cytoplasm that contained Periodic Acid–Schiff (PAS)-positive, Diastase-resistant (PAS-D) cytoplasmic zymogen granules, a feature of ACC^38^. Consistently, these lesions lacked mucin, Cytokeratin 17/19, and Sox9 expression, but were positive for the acinar markers, Amylase and Trypsin-2 (Fig. 4e, top panel). Likewise, in *Ptf1α*^*Cre*^*;Bap1*^*KO*^*;Kras*^*G12D*^ mice we observed small areas emulating ACC adjacent to PDA with glandular histology formed by well-differentiated cells in monolayers with basally located nuclei and eosinophilic cytoplasm (Fig. 4e bottom panel and Supplementary Fig. 4k). Although cells were variably PAS-D positive, they were negative for ductal and acinar markers, and may represent atypical manifestations, consistent with the finding that a small percentage of ACC stain negative for acinar markers^38,39^. The only patient diagnosed with ACC in the TCGA-PAAD cohort harbored heterozygous loss of *BAP1* but had wild-type *KRAS* and *TP53* (Fig. 4d and Supplementary Fig. 4l). These data suggest that BAP1 loss may also play a role in ACC.

### Loss of Bap1 causes genomic instability

**Fig. 5:**
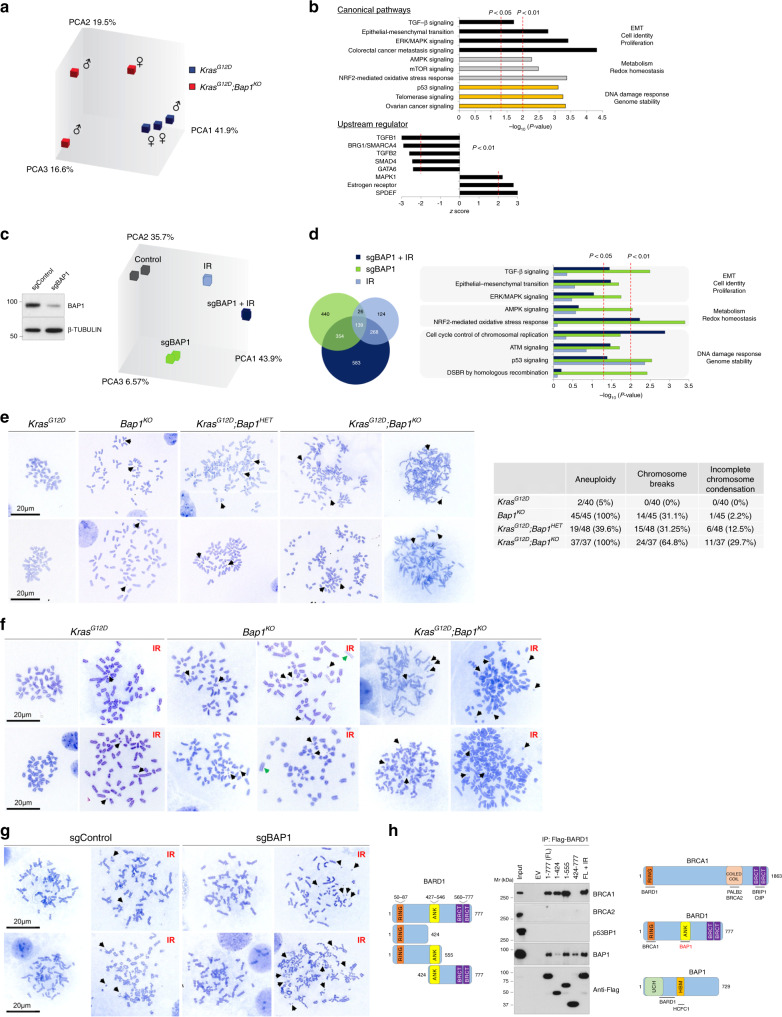
BAP1 regulates the DNA damage response. **a** Principal component analysis of transcriptomes of wild-type and *Bap1*-knockout cell lines established from the *Pdx1*^*Cre*^*;Kras*^*G12D*^ cohort. **b** Canonical pathway (top) and upstream regulator (bottom) IPA of DEG (fold change > 1.5 and *p* < 0.05) in *Bap1*-knockout cell lines. The *x* axis corresponds to the raw binomial *p*-values. **c** Left: western blotting showing BAP1 levels in PANC1 transfected with sgBAP1 or non-targeting guide sequence. Right: principal component analysis of transcriptomes of PANC1-treated with sgBAP1 before and 3 days after exposure to 10 Gy of IR. **d** Left: Venn diagram showing the overlap of DEG (fold change > 1.5 and *p* < 0.01) identified in PANC1 cells for the indicated conditions compared with non-treated control cells. Right: IPA of DEG for each condition compared to control. The *x* axis corresponds to the raw binomial *p*-values. **e** Representative metaphase spreads of murine pancreatic cancer cell lines of the indicated genotypes from the *Pdx1*^*Cre*^ cohort. *Bap1*^*KO*^*;Kras*^*G12D*^ cells show aneuploidy, chromosome shattering, and incompletely condensed chromosomes in the right panels. Right: table shows the number of metaphase nuclei with numerical and structural abnormalities. Results are representative from at least two independently established cell lines for each genotype. Black arrows point to chromosome fragments. **f** Representative metaphase spreads of murine pancreatic cell lines before and 3 days after exposure to 10 Gy of IR. Black and green arrows point to chromosome fragments and the absence of centromeres, respectively. **g** Representative metaphase spreads of HEK293T cells treated with sgBAP1 before and 3 days after exposure to 10 Gy of IR. Black arrows point to chromosome fragments. **h** HEK293T cells were transfected with Flag-tagged wild-type and the indicated truncated mutants of BARD1 (left). Cells expressing full-length (FL) BARD1 were also exposed to 10 Gy of IR (last lane). Nuclear extracts were immunoprecipitated with anti-Flag antibody and analyzed by western blotting for the indicated endogenous proteins. Right: schematic of the BRCA1, BARD1, and BAP1 proteins. UCH, ubiquitin carboxyl-terminal hydrolase; BRCT, BRCA1 C terminus; ANK, Ankyrin tandem repeats; HBM, HCF-1-binding domain. Not to scale.

To delineate the molecular pathways, we analyzed the transcriptomes of cell lines established from *Pdx1*^*Cre*^*;Kras*^*G12D*^ wild-type and *Bap1*-null mice. Consistent with the aggressive behavior of tumors in vivo, *Bap1*-knockout cell lines proliferated faster in vitro (Supplementary Fig. 5a). Affymetrix exon level analysis and western blotting confirmed the recombination of floxed exons and the absence of Bap1 protein (Supplementary Fig. 5b, c). The expression of PR-DUB and PRC1/2 members were unaltered (Supplementary Fig. 5c, d). Principal component and Ingenuity Pathway (IPA) analyses revealed pronounced changes in gene expression and enrichment for pathways regulating cell identity and proliferation (epithelial-to-mesenchymal transition, transforming growth factor-β (TGF-β), and mitogen-activated protein kinase signaling), metabolism and redox homeostasis (AMPK/mammalian target of rapamycin and NRF2 signaling), and DDR (p53, telomerase, and ovarian cancer signaling, which is frequently driven by mutations in BRCA1/2, Fig. 5a, b). Likewise, IPA inferred inhibition of TGF-β, BRG1/SMARCA4—a chromatin regulator frequently inactivated in IPMNs^40^, and GATA6—a transcription factor that regulates pancreatic fate^41^. Consistent with the cystic and mucinous histology of *Bap1*-null tumors, gene expression changes were concordant with activation of transcription factors that drive the maintenance of secretory epithelia such as estrogen receptor and SPDEF (SAM pointed domain containing ETS transcription factor)—a TGF-β-repressed transcription factor that regulates goblet cell differentiation^42^. Finally, expression of *Cftr* showed a more than tenfold downregulation in the Affymetrix arrays and was confirmed by quantitative reverse transcriptase-PCR in cell lines established from knockout pancreata (Supplementary Fig. 5e).

Next, we profiled PANC1 cells that were exposed to IR in the context of BAP1 downregulation (sgBAP1) mediated by CRISPR/Cas9. IR or sgBAP1 alone caused concordant changes in gene expression (~30% of differentially expressed genes, DEGs), whereas simultaneous perturbation had an additive effect (Fig. 5c, d). IPA showed enrichment for pathways regulating Epithelial-Mesenchymal Transition (EMT), metabolic and redox homeostasis, and DDR, in particular upon exposure to IR in the context of BAP1 deletion (Fig. 5d). Consistently, human and murine BAP1-deficient pancreatic cancer cell lines showed a pronounced sensitivity and activation of ATM/ATR kinases and H2A.XSer139 phosphorylation upon different means of DNA damage (Supplementary Fig. 5f, g). Although BAP1-knockout cells harbored increased H2AK119Ub, this modification was largely unaffected by DNA damage, suggesting the role of BAP1 in DDR may be independent of the deubiquitinase activity. Indeed, reconstitution of knockout cells with wild-type or a catalytically inactive mutant of BAP1 (BAP1^C91A^) revoked the sensitivity to IR (Supplementary Fig. 5h). We also observed that although sorted cells were expressing a similar amount of wild-type and mutant protein, BAP1 halted proliferation leading to counter-selection upon passaging (Supplementary Fig. 5i). In contrast, BAP1^C91A^ conferred a proliferative advantage and drove positive selection even when cells were plated at low densities. Mutations abolishing the deubiquitinase activity of BAP1 are not detected in PDA patients but are found in other malignancies and may confer an oncogenic potential. Interestingly, knockdown of *BAP1* in heterozygous SW1990 and PANC1 had the opposite effect and slightly reduced cell proliferation (Supplementary Fig. S5j). Given that both cell lines harbor additional defects in genes regulating DNA repair (Supplementary Fig. S1f), complete loss of *BAP1* may sensitize and render cells unable to cope with accumulation of excessive DNA damage.

DNA DSBs are lesions that must be repaired before cell division ensues. BAP1 is required for efficient HR^21,22^, a function that has been attributed to its interaction with BRCA1^43^. Indeed, by using a green fluorescent protein (GFP)-based reporter assay employing a recognition site for the rare-cutting I-SceI endonuclease for induction of DSBs^44^, we found that loss of BAP1 caused a substantial reduction of GFP-positive cells, indicating loss of proper DNA repair (Supplementary Fig. 5k). Consistently, metaphase chromosome spreads revealed a spectrum of abnormalities in knockout cells—even in the absence of oncogenic Kras—including chromosome breaks, shattering, premature sister chromatid separation with incompletely condensed chromosomes, and aneuploidy, which were exacerbated upon exposure to IR (Fig. 5e, f). *Kras*^*G12D*^ cells exhibited normal karyotypes that were disrupted upon BAP1 ablation. Deletion of BAP1 in HEK293T cells and exposure to IR also led to pronounced chromosomal abnormalities (Fig. 5g), suggesting an essential role in maintaining genome integrity. Furthermore, we detected a defective formation of PALB2 foci, a scaffold protein in the BRCA1–PALB2–BRCA2 complex, following DNA damage induced by IR. No alterations were observed in 53BP1 foci formation, a protein that regulates non-homologous end joining (Supplementary Fig. 5l). Although we failed to detect a direct interaction with BRCA1, we found that BAP1 directly interacts with the tandem ankyrin domains of BARD1 (BRCA1-associated RING domain 1) (Fig. 5h), a protein that interacts with BRCA1 to regulate DNA repair mediated by HR and protection of nascent DNA at stalled replication forks^45–47^.

### BAP1 regulates the response to DNA damage

**Fig. 6:**
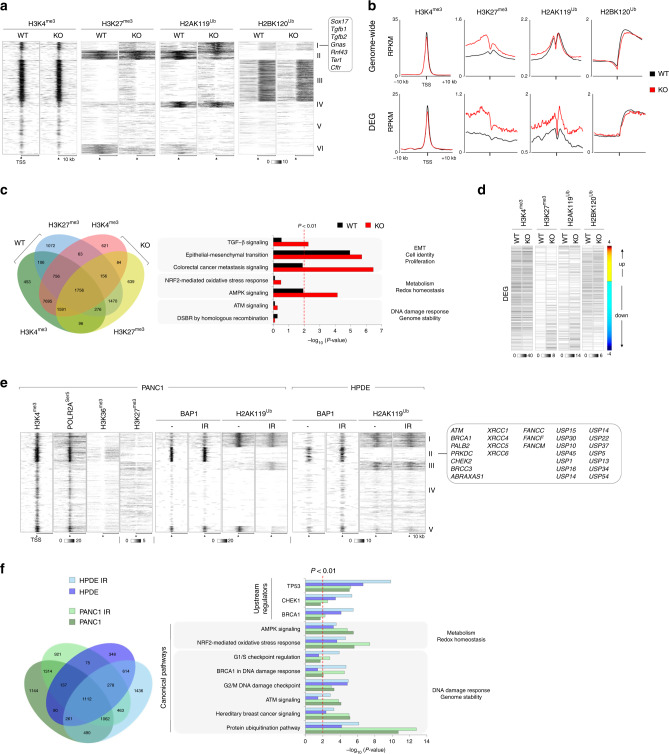
BAP1 binds active chromatin and regulates the response to DNA damage. **a** Composite heatmap showing the *K*-means clustering (*K* = 6) of the genome-wide distribution and signal intensity of the indicated histone modifications in *Pdx1*^*Cre*^*;Kras*^*G12D*^ wild-type and *Bap1*-knockout cells. Each horizontal line represents the normalized signal intensity for a gene over its transcription start site (TSS). A ±10 kb window is shown for each TSS. The grayscale bar shows the normalized signal intensity (RPKM, reads per kilobase per million mapped reads). **b** Read density profiles of the indicated histone modifications over the TSS ± 10 kb genome-wide (top) and DEG (bottom) in *Pdx1*^*Cre*^*;Kras*^*G12D*^ wild-type and *Bap1*-knockout cells. The *y* axis shows the mean RPKM. **c** Left: Venn diagram showing the overlap of the indicated histone modifications in *Pdx1*^*Cre*^*;Kras*^*G12D*^ wild-type and *Bap1*-knockout cells. Right: IPA of canonical pathways linked to de novo bivalent genes in *Bap1*-knockout cells. The *x* axis corresponds to the raw binomial *p*-values. **d** Heatmap showing the signal intensity of the indicated histone modifications over the DEG (fold change > 1.4 and *p* < 0.05) in *Pdx1*^*Cre*^*;Kras*^*G12D*^ wild-type and *Bap1*-knockout cells. The color bar represents DEG sorted based on the fold-change difference in gene expression between knockout and wild-type cells. **e** Composite heatmap showing the *K*-means clustering (*K* = 5) of the genome-wide distribution and signal intensity of the indicated histone modifications, POLR2A^Ser5^, and BAP1 in PANC1 and HPDE before and 1 h after exposure to 10 Gy of IR. The grayscale bars show the normalized RPKM. **f** Left: Venn diagram showing the overlap of genes bound by BAP1 in HPDE and PANC1 before and after IR (10 Gy). Right: canonical and upstream regulator IPA of BAP1-bound genes in HPDE and PANC1 in response to IR. The *x* axis corresponds to the raw binomial *p*-values.

To systematically study and causatively link epigenetic alterations with gene expression changes, we mapped the distribution of H3K4me3, H3K27me3, H2AK119Ub, and H2K120Ub in wild-type and knockout cells. Peak calling followed by *K*-means clustering and genome-wide pairwise comparison of the normalized signal intensity over the transcription start site (TSS) revealed increases of repressive H2AK119Ub and H3K27me3 (cluster I in Fig. 6a and Supplementary Fig. 6a), particularly over DEGs (Fig. 6b). Besides genes driving pancreas cell fate (*Sox17*), Cluster I also contained members of TGF-β (*Tgfb1/2*) and genes (*Gnas* and *Rnf43*) frequently deregulated in IPMNs and MCNs, as well as *Cftr*. In contrast, no significant alterations were observed in the distribution and signal intensity of H3K4me3 and H2BK120Ub, nor in H3K4me1 and H3K27ac that define poised and active super-enhancers, respectively (Supplementary Fig. 6b). Multiple promoters with a gain of H3K27me3 in knockout cells were pre-marked with H3K4me3 and the de novo bivalent genes were enriched for pathways regulating cell-fate decisions and metabolic homeostasis, but not DDR (Fig. 6c). Loss of BAP1 increased the intensity of both H2AK119Ub and H3K27me3, and repressed the majority of DEG (66.2% downregulated vs. 33.8% upregulated, Fig. 6d). Hence, BAP1 primarily functions as a transcriptional coactivator by removing repressive H2AK119Ub.

Next, we mapped the genome-wide binding of BAP1 and distribution of H2AK119Ub in PANC1, which exhibits heterozygous loss of *BAP1* (Supplementary Fig. 1e), as well as in immortal, but not transformed, human pancreatic ductal endothelial (HPDE) cells, in response to IR. *K*-means clustering revealed that BAP1 binds active chromatin enriched for H3K4me3, H3K36me3, and active RNA polymerase II phosphorylated at Ser5 (POLR2ASer5), but is depleted for repressive H3K27me3 and H2AK119Ub (cluster II in Fig. 6e and Supplementary Fig. 6c). BAP1 and H2AK119Ub signal intensities were highly concordant in the two cell lines and largely unaffected by IR (Supplementary Fig. 6d, e), further supporting that gene expression changes triggered by IR are independent of the catalytic activity of BAP1. Consistently, IPA of genes bound by BAP1 showed significant overlap between PANC1 and HPDE, and enrichment for pathways and upstream regulators of DDR such as protein ubiquitination, ATM, and BRCA1 signaling pathways among others, particularly in response to DNA damage (Fig. 6e, f).

### BAP1 loss confers sensitivity to DNA damage treatments

**Fig. 7:**
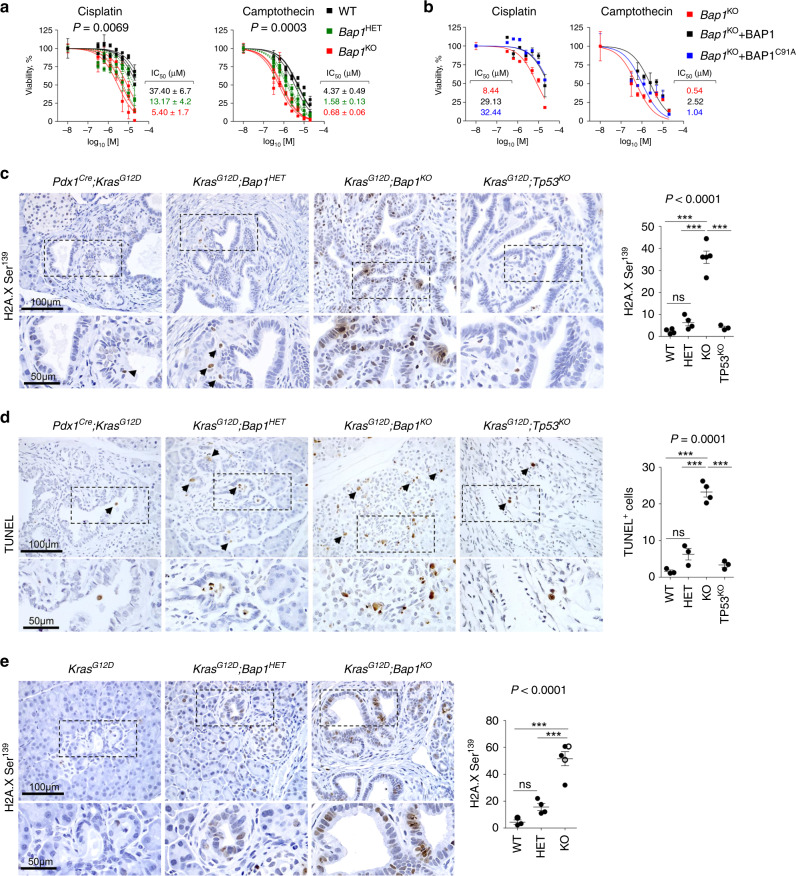
BAP1 deficiency confers radio- and chemo-sensitivity. **a** Estimation of IC_50_ values for cisplatin (left) and camptothecin (right) for wild-type (*n* = 3), heterozygous (*n* = 3), and *Bap1*-knockout (*n* = 3) pancreatic cell lines independently established from the *Pdx1*^*Cre*^*;Kras*^*G12D*^ cohort and assessed in triplicates. The average IC_50_ values (mean ± SEM) are shown. Statistical significance was determined by one-way ANOVA with *p*-values shown on the top of each plot. **b** Estimation of IC_50_ values for cisplatin (left) and camptothecin (right) for a *Bap1*-knockout pancreas cell line reconstituted with wild-type and BAP1^C91A^ mutant. The graphs show cell viability assessed in triplicates (mean ± SEM) for the indicated concentrations of compounds. **c**, **d** 10–15-week-old mice of the indicated genotypes were exposed to 10 Gy of IR. Three days later, mice were killed and pancreata were stained for (**c**) H2AXSer139 (WT *n* = 4, HET *n* = 4, KO *n* = 5, and TP53^KO^ *n* = 3 mice) and (**d**) TUNEL (WT *n* = 3, HET *n* = 3, KO *n* = 4, and TP53^KO^ *n* = 3 mice). **e** 6–10-week-old mice of the indicated genotype (WT *n* = 3, HET *n* = 4, and KO *n* = 5 mice) were treated weekly for 4 weeks with 5 mg/kg cisplatin. Mice were killed and pancreata were stained for H2AXSer139. In **c–e**, the scatter dot plots show the number of positive cells (mean ± SEM) per 0.1 mm^2^ of tissue per mouse. Each dot represents a mouse. Black arrows point to positively stained cells. Filled and open circles indicate mice from the *Pdx1*^*Cre*^ and *Ptf1α*^*Cre*^ cohorts, respectively. Statistical significance was determined by one-way ANOVA with *p*-values shown on the top of each plot, followed by Tukey’s multiple comparison post-hoc test between groups. ns, nonsignificant; ***p < 0.001. Source data are provided as a Source Data file.

To test the response to DNA damage, murine wild-type, heterozygous, and knockout cells as well as human PDA cell lines treated with shBAP1 were exposed to cisplatin (a DNA cross-linker) and camptothecin (topoisomerase I inhibitor). We found that loss of BAP1 conferred an increased sensitivity (Fig. 7a and Supplementary Fig. 7a). Reconstitution of knockout cells with wild-type or mutant BAP1^C91A^ abolished sensitivity, suggesting a deubiquitinase independent function (Fig. 7b). On the other hand, knockout cells were resistant to gemcitabine, a nucleoside analog that is commonly used for treating pancreatic cancer (Supplementary Fig. 7b). Meta-analysis of pharmacogenomic data from the Cancer Cell Line Encyclopedia through the CellMinerCDB portal^48^ revealed that low BAP1 expression correlated with sensitivity to irinotecan (derivative of camptothecin) specifically in human pancreatic cancer cell lines and linear regression analysis among DNA repair genes highlighted BAP1 in explaining the response (Supplementary Fig. 7c, d). Similarly, BAP1-null mouse and human pancreatic cancer cell lines were sensitive to IR and failed to form colonies when plated at low densities (Supplementary Fig. 7e). Unlike BRCA1/2 mutant tumors^49^, BAP1-null pancreatic cancer was resistant to PARP inhibitors (Supplementary Fig. 7f). Likewise, no sensitivity was detected to compounds targeting the PRC2 (Supplementary Fig. 7g, h).

To confirm these findings in vivo, *Kras*^*G12D*^*;Bap1*^*KO*^ mice were exposed to IR and analyzed for markers indicative of DNA damage and apoptosis. Unlike *Kras*^*G12D*^ or *Kras*^*G12D*^*;Trp53*^*KO*^ mice where IR had a minor effect, *Kras*^*G12D*^*;Bap1* heterozygous and knockout pancreata showed a striking increase in the number and intensity of malignant cells staining positive for H2A.XSer139 (Fig. 7c). TUNEL and IHC for cleaved Caspase-3 confirmed an increase in apoptotic cells, selectively in *Bap1*-knockout tumors (Fig. 7d and Supplementary Fig. 7i). Similar results were obtained in mice treated with cisplatin (Fig. 7e), suggesting that loss of BAP1 confers sensitivity to IR and platinum-based chemotherapy.

## Discussion

Chronic pancreatitis is a risk factor for the development of pancreatic cancer and has been linked to alcohol intake, smoking, and poorly defined genetic factors^31^. Unlike hereditary pancreatitis due to mutations in genes that regulate exocrine pancreas homeostasis, such as *CFTR*, somatic CNAs that predispose to chronic inflammation and pancreatitis remain unknown. We found that loss of BAP1 leads to the development of chronic pancreatitis in mice and is associated with poor prognosis in PDA patients. Notably, *Brca2* inactivation also promoted an inflammatory response and disrupted exocrine pancreas homeostasis in mice^9^, and *BRCA1* downregulation has been documented in patients diagnosed with chronic pancreatitis^50^. Likewise, DNA repair genes, including *Bap1*, are readily upregulated and required for pancreas regeneration in caerulein-treated mice. Although further proof is needed, these data suggest that defective DDR may be a common denominator in a subset of chronic pancreatitis cases and causatively linked to a higher risk for PDA.

Individuals with germline mutations in DNA repair genes have a higher risk of developing PDA^17,51^. Although over 80% of familial pancreatic cancer cases harbor mutations in *BRCA1/2* and *ATM*, germline deletion and mutations of *BAP1* have also been observed^13,15–18^. On the other hand, somatic mutations of DNA repair genes are rare (<5% of patients), which is counterintuitive given that recent sequencing studies showed a transcriptional signature of defective DDR in about 25% of PDA patients^2,4^. We found that somatic heterozygous loss of *BAP1* may account for this discrepancy, as it is the most frequent CNA among DNA repair genes occurring in over a quarter of PDA and 40% of ACC^24,52,53^. Although LOH of additional genes within Chr 3p21.1 may contribute to tumor suppression, and loss of *BAP1* in pancreatic cancer may be secondary—rather than the cause of genomic instability—our mouse model suggests *Bap1* ablation suffices to cause defective DNA repair, pancreatitis, and cooperates with oncogenic *Kras* to promote pancreatic cancer. Thus, downregulation of *BAP1* alleviates a barrier to pancreatic cancer progression, but at the same time its low expression maintains basal DNA repair mechanisms protecting rapidly dividing cancer cells from the catastrophic consequences of uncontrolled accumulation of DNA damage.

Although deletion of *Bap1* in the context of *Kras*^*G12D*^ induced MCNs and IPMNs, in *Ptf1α*^*Cre*^-knockout pancreata we also observed areas with features of acinar cell transformation. Although not yet comprehensive, human ACC exhibits frequent loss for *BAP1* and other DNA repair genes in more than half of the cases^24,52^. Acinar tumors have been reported in murine pancreata with biallelic *Brca2* mutations^9^. Altogether, defective DDR may drive ACC and simultaneous inactivation of multiple genes involved in HR may be required to phenocopy this rare pancreatic cancer subtype accurately.

Defining biomarkers of responsiveness to chemotherapy and IR can significantly improve treatment and clinical outcomes. BAP1-deficient pancreatic cancer showed numerical and structural chromosomal abnormalities and sensitivity to IR and compounds that cause replication fork stalling, such as cisplatin and camptothecin. Derivatives of cisplatin and camptothecin (oxaliplatin and irinotecan, respectively) are components of FOLFIRINOX which is emerging as a first-line treatment for pancreatic cancer^11^, whereas oxaliplatin showed efficacy as a single agent in ACC^54^. Although defective DDR is causative and permissive of pancreas oncogenesis, it may also uncover an Achilles heel to be exploited therapeutically. Mechanistically, BAP1 regulates the repair of DNA DSBs by HR and directly interacts with BARD1, a protein that forms a heterodimer with BRCA1 to regulate HR and stalled fork protection^45–47^. The latter is crucial in protecting stalled DNA replication forks in rapidly dividing cancer cells from nucleolytic degradation and may explain the sensitivity to platinum-based compounds and topoisomerase inhibitors^55,56^. Given that heterozygous loss of *BAP1* is the most frequent somatic aberration among DNA repair genes in pancreatic cancer, our findings establish a rationale for evaluating BAP1 status to stratify patients who are likely to respond to FOLFIRINOX and radiotherapy.

## Methods

### Study approval

Mouse experiments were conducted under protocols A292, A293, and A308, which were reviewed and approved by the Institutional Animal Care and Use Committee of the George Washington University, Washington DC. Commercially available de-identified human TMAs were bought from the Cooperative Human Tissue Network (CHTN, which is funded by the National Cancer Institute; www.chtn.org/) and Biomax (www.biomax.us/). The CHTN_PancProg1 TMA contains a series of normal, premalignant (low- and high-grade PanIN), pancreatic carcinoma, and metastatic specimens. The Biomax TMAs (PA501 and PA1921a) contained specimens from normal and clinically annotated de-identified pancreatic cancer patients. Redundant patient specimens among the Biomax TMA employed were excluded from the analysis. According to the guidelines from the Office of Human Research Protection, the conducted research meets the criteria for exemption #4 [45 CFR 46.101 (b) Categories of Exempt Human Subjects Research] and does not constitute human research.

### Animal studies

*Bap1*^*tm1a(EUCOMM)Hmgu*^ (C57BL/6NTac-Bap1 < tm1a(EUCOMM) Hmgu > /Wtsi) mutant mice were developed by the Wellcome Trust Sanger Institute Mouse Genetics Project based on the “knockout-first allele” strategy^27^. To generate pancreas-specific reporter (*Bap1*^*lacZ/+*^), mice expressing β-galactosidase under the endogenous *Bap1* promoter mice were crossed with the *Pdx1*^*Cre*^^28^ strain. To generate conditional knockout animals, *Bap1*^*lacZ/+*^ mice were sequentially crossed with *β-Actin*^*FLPe*^^57^ to remove the *Frt* cassette and the offspring to *Pdx1*^*Cre*^ and *Ptf1α*^*Cre*^^29^ strains to delete *Bap1* in pancreatic progenitor and acinar cells, respectively. To generate the experimental cohorts, *Pdx1*^*Cre*^ or *Ptf1α*^*Cre*^ *Bap1* and *Trp53*^58^ heterozygous mice were crossed to *LSL-Kras*^*G12D/+*^^59^ strain. Mice were bred on a mixed C57BL/6;129/Sv background. Genotyping primers for *Bap1*^*tm1a(EUCOMM)Hmgu*^ mice are shown in Supplementary Table 1. To induce pancreatitis, mice were intraperitoneally injected for two consecutive days with 400 μg/kg/day caerulein (Sigma-Aldrich) in phosphate-buffered saline (PBS) divided into four doses/day and administered every 2 h. Mice were analyzed 5 days post caerulein treatment, a time point where wild-type pancreata are expected to have been almost fully regenerated. To induce DNA damage, mice were exposed to 10 Gy IR (^137^Cs source) and killed 3 days later for analysis. Treatment of mice with cisplatin (5 mg/kg) in PBS occurred intraperitoneally, once per week, starting at 5–6 weeks of age and lasted 4 weeks. In all experiments, littermate pairs were used.

### Immunohistochemistry

Pancreata and liver were formalin fixed, paraffin embedded, and sectioned on a Leica RM2165 microtome. Four-micrometer tissue sections were de-paraffinized in xylenes, rehydrated sequentially in ethanol, washed in 0.5% Triton X-100 in PBS, rinsed in water, and submerged in citrate buffer (pH 6.0) to unmask antigens using the Retriever 2100 (Aptum), according to manufacturer’s protocol. Then, slides were incubated with 3% H_2_O_2_ to block endogenous peroxidase activity, washed, and blocked with 10% normal horse serum for 1 h. Primary antibodies were applied overnight at 4 °C. The next day, slides were washed, incubated with biotinylated secondary antibodies for 1 h at room temperature, treated with VECTASTAIN^®^ Elite ABC-HRP Kit for 30 min, developed with the DAB substrate kit (Vector Laboratories), counterstained with hematoxylin, and mounted with Dibutylphthalate Polystyrene Xylene (DPX). For BAP1 IHC, Buffer B (pH 8.0) was used for antigen retrieval. For MUC2 and MUC5AC IHC, the Vector M.O.M. Basic Immunodetection kit (Vector Laboratories) was used according to the manufacturer’s protocol. For Sirius Red staining, slides were placed flat in a chamber, 200–300 μl of Sirius Red/Fast green stain was added to cover tissues, and stained for 30 min. Slides were thoroughly rinsed in water and briefly dehydrated through sequential alcohols, cleared in xylenes, and mounted with DPX. The PAS-D staining was performed at the research pathology core lab of George Washington University (https://smhs.gwu.edu/pcl/). Scoring for BAP1 nuclear staining was based on the intensity as “absent, weak, or strong” and determined with the Zen lite 2012 software. The chi-square test was used to determine whether there was a significant difference between the expected and observed frequencies in one or more categories. Supplementary Table 2 provides the catalog numbers and dilutions of antibodies.

### X-gal tissue staining

Pancreata were rinsed in PBS and fixed in 4% paraformaldehyde for 1 h at room temperature, then washed once with PBS and twice in staining buffer (2 mmol/L MgCl_2_, 0.01% Na-Deoxycholate, 0.02% NP-40 in PBS) for 15 min each at room temperature. Pancreata were stained overnight at 37 °C in X-gal staining solution (1 mg/ml X-gal, 5 mmol/L K_4_Fe(CN)_6_, and 5 mmol/L K_3_Fe(CN)_6_ in staining buffer), followed by three washes in PBS for 15 min each, fixed in 10% neutral buffered formalin overnight, and paraffin embedded. Four-micrometer sections were de-paraffinized in two changes of xylenes and rehydrated sequentially in ethanol. Sections were counterstained in nuclear fast red (Amresco), dehydrated sequentially in ethanol, cleared in two changes of xylenes, and mounted with DPX.

### TUNEL assay

Click-iT™ TUNEL Colorimetric IHC Detection Kit (Invitrogen) was used for detecting cell apoptosis according to the manufacturer’s protocol. Briefly, pancreatic tissue sections were de‐paraffinized, rehydrated sequentially, washed with PBS, fixed at room temperature in 4% paraformaldehyde for 15 min, digested with proteinase K for 15 min, and finally fixed at room temperature in 4% paraformaldehyde for 5 min. The sections were incubated with the TdT reaction mixture at 37 °C for 1 h in a humidity chamber. The TdT reaction was quenched by incubating the slides in 2× Saline Sodium Citrate (SSC) buffer for 15 min and endogenous peroxidase activity was blocked by incubating the slides in 3% hydrogen peroxide for 5 min. The slides were then incubated in the TUNEL Colorimetric reaction cocktail at 37 °C for 30 min in a humidity chamber, in the dark. Next, the slides were incubated with a Streptavidin-Peroxidase Conjugate for 30 min at room temperature in a humidity chamber. After developing with DAB, slides were counterstained in hematoxylin, dehydrated sequentially in ethanol, then cleared in two changes of xylenes, and mounted with DPX. Nuclei of cells stained brown were regarded as positive apoptotic cells by TUNEL staining.

### Histomorphometry

All slides were photographed with a Zeiss AxioLab.A1 and AxioCam ICc5 camera, and analyzed with the Zen lite 2012 software as indicated in the figure legends. To quantify the number of cells responding to different treatments sections were photographed at ×20 or ×40 magnification as indicated in the figure legends, the tumor area was delimited with the ‘spline contour’’ tool of the Zen lite 2012 and calculated by the software. Data are presented as the number of positive cells per tumor area (mm^2^) ± SEM. The ‘spline contour’’ tool was also employed to quantify the area of pancreas with signs of pancreatitis.

### Glucose, amylase, and lipase measurements

Blood glucose levels (mg/dl) were quantified using the Bayer Contour Next One Glucose Meter. Mice were either fed ad libitum or fasted overnight. Levels of amylase and lipase in the blood were measured using as reference the ethylidenep-NP-G7 (catalog # MAK009, Sigma-Aldrich) and glycerol (catalog # MAK046, Sigma-Aldrich) substrates in kinetic activity assays, respectively. Serum (2 μL) was diluted in to 1 : 1 ratio with normal saline and mixed with either the amylase or lipase assay buffer to initiate the reaction. The increase in absorbance due to the release of *p*-nitrophenol and glycerol was monitored at 405 nm and 570 nm for amylase and lipase, respectively. Activity was recorded as nmole/min/ml and reported as Units/Liter (U/L). One unit of amylase is the amount of the enzyme that cleaves ethylidene-pNP-G7 to generate 1.0 μmol of *p*-nitrophenol per minute at 25 °C. One unit of lipase is the amount of enzyme that will generate 1.0 μmol of glycerol from triglycerides per minute at 37 °C.

### Human and murine pancreatic cancer cell lines

Human pancreatic cancer cell lines were bought from American Type Culture Collection and maintained in Dulbecco’s modified Eagle’s medium (DMEM) supplemented with 10% (vol/vol) cosmic calf serum, 4 mM l-glutamine, 1 mM sodium pyruvate, and 1% (vol/vol) penicillin/streptomycin^60,61^. For murine cell lines, tumor-bearing pancreata were minced, collagenase digested, and single-cell suspensions were plated on collagen-coated (rat tail type I) plates^60,61^. Primary murine cell lines were maintained in DMEM/F12 without phenol red, 5 mg/ml d-Glucose, 1.22 mg/ml Nicotinamide, 100 ng/ml Cholera toxin, 5 ml/L Insulin-Transferrin-Selenium Plus, 0.1 mg/ml soybean trypsin inhibitor, 20 ng/ml epidermal growth factor, 5% Nu-Serum IV culture supplement, 25 μg/ml bovine pituitary extract, 5 nM 3,3,5-tri-iodo-l-thyronine, 1 μM dexamethasone, 1% Pen/Strep, and plasmocin (Invivogen). Two to three weeks later, collagen was removed and cells were expanded in 75% pancreatic media/25% complete DMEM. Early passage cell lines were used for all in-vitro experiments. For proliferation assays, 5 × 10^4^ cells were plated in duplicate or triplicate in 12-well plates and passaged to 1 : 2 or 1 : 4 ratio. Cells were counted with a Bio-Rad TC20 automatic cell counter. For reconstitution of murine *Bap1*-knockout pancreatic cancer cell lines retroviruses encoding BAP1-WT or BAP1-C91A (Addgene, #81024 and #81025, respectively^62^) were generated by transfecting HEK293T cells with the corresponding constructs and viral supernatants were collected after 2 days and used for subsequent infections. Transduced cells were sorted (BD Influx) for the cell surface marker Thy1.1 (Biolegend, clone OX-7) and expanded for subsequent experiments. The Flag-BARD1^63^, GFP-tagged PALB2, and p53BP1 (Addgene #71113 and #60813^64,65^) expression vectors have been previously described. To knock down BAP1, cells were infected with lentiviruses expressing short-hairpin RNAs cloned in the pLKO.1 puromycin vector developed by the RNAi Consortium (TRC, Broad Institute). The following short hairpins targeting human *BAP1* were used: TRCN0000007372 5′-CGGCGTGGAAGATTTCGGTGTCAACTCGAGTTGACA CCGAAATCTTCCACGTTTTT-3′ and TRCN0000007374 5′-CCGGCCACAACTACGATGAGTTCATCTCG AGATGAACTCATCGTAGTTGTGGTTTTT-3′. A pLKO.1-puromycin vector encoding an shRNA for luciferase (shLUC) was used as control. To delete *BAP1* in human cells, we employed the CRISPR/Cas9 technology and guide RNA (5′-TACCGAAATCTTCCACGAGC-3′) targeting exon 2 or non-targeting guide control were cloned in the pX459-V2 vector (Addgene #62988). After transfection, cells were selected with puromycin and genomic DNA was isolated and sequenced for the presence of indels in the genomic locus of *BAP1* followed by western blotting to confirm the absence of BAP1 protein.

### Flow cytometry

Pancreata were minced and digested in 5 ml DMEM/F12 (without phenol red and supplemented with 5 mg/ml d-glucose) in the presence of collagenase IV (1 mg/ml, StemCell Technologies #07909), dispase (1 U/ml, StemCell Technologies #07923), hyaluronidase (100 U/ml, Worthington Biochemical), and DNase type I (100 U/ml, Sigma-Aldrich) for 2–3 h at 37 °C with periodic mixing. Dissociated cells were washed twice in PBS prior to filtering out debris using a 70 μm mesh filter. Single cells were resuspended in flow staining buffer (5% horse serum in PBS) and stained with fluorochrome-conjugated antibodies against T-cell marker CD3e (BV-421; clone 145-2C11), B cell marker B220 (APC; clone RA3-6B2), dendritic cell marker CD11c (PE; clone N418), and macrophage cell marker CD11b (FITC; clone M1/70). Cell viability was assessed with SYTOX™ Blue Dead Cell Stain (ThermoFisher #S34857). Data collection took place on a 3-laser BD Celesta analyzer and analysis was performed with FlowJo software (TreeStar). Supplementary Table 2 provides the catalog numbers and dilutions of antibodies.

### Western blotting and immunoprecipitation

Cells were solubilized on ice in lysis buffer (25 mM Tris-HCl pH 7.6, 200 mM NaCl, 1 mM EDTA, 1% Igepal CA-630, 0.1% Na-Deoxycholate, 0.1% SDS supplemented with a mixture of protease and phosphatase inhibitors), centrifuged for 10 min at 16,000 × *g*, and the supernatant was analyzed by western blotting. For immunoprecipitation, cells were incubated with hypotonic buffer A (20 mM HEPES, 10 mM KCl, 1 mM MgCl_2_) for 10 min followed by centrifugation at 2000 × *g* for 5 min at 4 °C. Pelleted nuclei were resuspended in buffer C (20 mM HEPES, 480 mM KCl, 0.1% Igepal CA-630, 0.1% SDS), incubated on ice for 20 min, briefly sonicated, and cleared by centrifugation at 16,000 × *g* for 10 min. Nuclear extracts were mixed with 30 μl of anti-FLAG beads (Sigma) and incubated overnight at 4 °C with gentle agitation. Immunoprecipitated materials were extensively washed, eluted with 2× loading buffer at 95 °C for 5 min, and analyzed by western blotting. Supplementary Table 2 provides the catalog numbers and dilutions of antibodies.

### Preparation of metaphase spreads and G-banding

To sequester cells in metaphase, colcemid (0.02 μg/ml final concentration, Sigma-Aldrich, St. Louis, MO) was added to the medium of cells in exponential growth and cells were incubated at 37 °C for 90 min. After incubation, cells were trypsinized, washed, and resuspended in hypotonic solution (75 mM KCl). Once in hypotonic solution, cells were then incubated at 37 °C for another 15 min. Swollen cells were centrifuged at 1000 × *g* for 8 min at 4 °C, aspirated, gently resuspended in 1 ml of fixative (3 : 1 methanol : glacial acetic acid), and incubated on ice for 30 min. Cells were centrifuged (1000 × *g* for 5 min at 4 °C), aspirated and resuspended in 1 ml fixative twice more. The final cell suspension was dropped onto an ethanol-cleaned slide and baked at 50 °C for 2 h. For G-banding, 1 week later, slides were incubated in 2 ×SSC (300 mM NaCl, 34 mM trisodium citrate) at 65 °C for 1.5 h. Slides were rinsed in 0.9% NaCl and stained with a Giemsa-Trypsin solution (1.25 μg of trypsin in 200 μL Hank’s buffered saline solution (137 mM NaCl, 5.4 mM KCl, 441 μM KH_2_PO_4_, 5.6 mM glucose, 352 μM Na_2_HPO_4_, 4 mM NaHCO_3_) diluted into 45 mL of 1 : 30 May-Grunwald Giemsa : Gurr’s Buffer (2 : 1 KH_2_PO_4_ (4 mM) : Na_2_HPO_4_ (4 mM), pH 6.8)) solution. The slides were rinsed in two changes of 1 : 1 Gurr’s Buffer : ddH_2_O, air dried, mounted with DPX, and photographed at ×63 with oil-immersion on a Zeiss AxioLab.A1.

### Homologous recombination repair assay

Wild-type and BAP1-knockout cells were transfected in duplicate with plasmids expressing the HR substrate composed of two differentially mutated GFP genes oriented as direct repeats (pDRGFP; Addgene #26475) and I-SceI endonuclease expression vector (pCBASceI; Addgene #26477). Expression of I-SceI endonuclease generates a site-specific DSB between the mutated GFP genes, which, when repaired by gene conversion, results in a functional GFP gene. Two days later, cells were collected for flow cytometry in a BD FACSCelesta to determine the percentage of GFP-positive cells. Data analyses were performed with FlowJo software (TreeStar). To adjust for different transfection efficiencies between cell lines, data were normalized by using a GFP reporter plasmid transfected in parallel.

### Gene expression analysis

Total RNA was isolated using the RNeasy spin column kit (Qiagen) and quantified using a BioSpectrometer (Eppendorf). One hundred nanograms of RNA was used as input for the GeneChip® PrimeView™ (3′ IVT human array) and GeneChip™ Mouse Gene 2.0 ST (mouse exon array) microarrays (Affymetrix). Synthesis, labeling, and purification of biotinylated complementary RNA and sense-stranded DNA targets were carried out according to manufacturers’ instructions using the GeneChip™ 3′ IVT PLUS Reagent Kit and GeneChip™ WT PLUS Reagent Kit, respectively. Hybridization was performed using a GeneChip Hybridization Oven 640 overnight at 45 °C. Microarray washing and staining was performed on a GeneChip Fluidics Station 450 and scanning on a GeneChip Scanner 3000 7G, commanded by the Affymetrix GeneChip Command Console software. Probe-level analysis including background subtraction and quantile normalization took place with the Robust Multi Array Average Algorithm using the Affymetrix Expression Console Software 1.3. DEGs (*p* < 0.05 and fold change > 1.5 for the GeneChip™ Mouse Gene 2.0 ST exon array and *p* < 0.01 and fold change > 1.5 for the GeneChip® PrimeView™) were determined using the Transcriptome Analysis Console v3.0. Raw and processed Affymetrix data have been deposited in the Gene Expression Omnibus repository under accession number GSE120127.

### Ingenuity Pathway analysis

DEGs were used as input for IPA to infer canonical pathways and upstream regulators that could explain gene expression changes caused by loss of BAP1 for *z*-scores >2 and a *p* overlap value < 0.01. The positive or negative sign of *z*-score indicates activation or repression, respectively, of a particular canonical pathway or upstream regulator in the context of BAP1 loss. A Fisher’s exact test was used to determine the statistical significance.

### Chromatin immunoprecipitation

Cells (1 x 10^7^) were cross-linked in 1% formaldehyde for 20 min, quenched with 125 mM glycine for 5 min, and lysed in ice-cold 0.5% Igepal CA-630 in PBS supplemented with protease inhibitors for 10 min on ice. Nuclei were pelleted, digested with 1 μl micrococcal nuclease for 3 min at 37 °C to fragment chromatin, resuspended in SDS lysis buffer (50 mM Tris-HCl pH 8.0, 1% SDS, 150 mM NaCl, and 5 mM EDTA), and briefly sonicated. Cells were centrifuged 16,000 × *g* for 10 min at 4 °C, and ten volumes of dilution buffer (16.7 mM Tris pH 8.0, 167 mM NaCl, 0.01% SDS, 1.1% Triton X-100, and 1.2 mM EDTA) was added to the soluble fraction. Chromatin immunoprecipitation (ChIP-Seq) took place on protein A agarose beads with antibodies against H3K4me1, H3K4me3, H3K27me3, H3K27ac, H2AK119ub, H2BK120ub, and BAP1 (Cell Signaling, Supplementary Table 2) overnight at 4 °C. Beads were washed twice for 10 min each with: low-salt buffer (20 mM Tris-HCl pH 8.0, 150 mM NaCl, 0.1% SDS, 1% Triton X-100, and 2 mM EDTA), high-salt buffer (20 mM Tris-HCl pH 8.0, 500 mM NaCl, 0.1% SDS, 1% Triton X-100, and 2 mM EDTA), LiCl buffer (10 mM Tris-HCl pH 8.0, 0.25 M LiCl, 1% Igepal CA-630, 1% Na-Deoxycholate, and 1 mM EDTA), and TE (10 mM Tris pH 7.6, 1 mM EDTA). DNA–protein cross-links were eluted with elution buffer (0.1 M NaHCO_3_, 1% SDS) at room temperature for 30 min. Reverse crosslinking was performed overnight at 65 °C by increasing the concentration of NaCl. Proteins were digested with proteinase K for 1 h at 42 °C and cleaning of the eluates took place with the QIAquick PCR Purification Kit (Qiagen). Libraries were prepared with the NEBNext Ultra^TM^ II DNA Library Prep kit for Illumina (New England BioLabs) and sequenced on an Illumina NextSeq 2000 instrument.

### ChIP-Seq data analysis

The FastQC (Version 0.10.1) software was used to examine the read quality. Reads were aligned to the mouse (mm9) or human (GRCh37/hg19) genome using Bowtie (Version 1.1.1^66^) with default parameters and keeping only uniquely aligned reads. SICER (Version 1.1^67^) was used for peak calling to identify chromatin domains enriched for BAP1 binding and histone modification marks. Redundant reads, which might be the result of PCR artifacts, were filtered before peak calling. Window size = 200 bp and gap size = 200 bp was used for H3K27ac, H3K4me3, and BAP1, and window size = 200 bp and gap size = 600 bp for H3K27me3, H3K4me1, H2AK119Ub, H2BK120Ub. False discovery rate threshold was set to 1e − 8 for all histone modifications and 1e − 5 for BAP1 ChIP. For peak annotation, we employed the RefSeq transcripts from the UCSC genome browser with a ±2 kb window spanning the TSS. Genome-wide comparison and *K*-means clustering of signal intensity as well as plotting the read density profile over the TSS and SE took place with EaSeq^68^ software. Island-filtered reads from SICER were used as input. For calling super-enhancers, we employed ROSE (Rank Ordering of Super-Enhancers)^69^ software using H3K27ac ChIP-Seq signal as input and default parameters. The GREAT (version 3.0.0) online tool was used to identify genes linked to SE based on the association rule: Basal+extension: 1 kb upstream, 1 kb downstream, 1000 kb max extension. Raw and processed data from ChIP-seq experiments have been deposited in the Gene Expression Omnibus repository under accession numbers GSE121709 and GSE120460. Raw ChIP-seq data for H3K4me3 (ENCFF346XSZ), POLR2ASer5 (ENCFF065NPZ), H3K36me3 (ENCFF356QYS), and H3K27me3 (ENCFF072NKY) for PANC1 were downloaded from ENCODE (https://www.encodeproject.org/).

### Induction of DNA damage and drug sensitivity

Cells were exposed to 10 Gy of IR (^137^Cs source), camptothecin (topoisomerase I inhibitor), cisplatin (DNA crosslinking agent), gemcitabine, and PARP inhibitors (olaparib, 3-ABA, and PJ-34) as indicated in the figure legends. To inhibit the PRC2 complex we employed EZH2 inhibitors GSK126 and GSK343, as well as EED inhibitors EED226 and A-395. All inhibitors were bought from Selleck (https://www.selleckchem.com/), except A-395, which was kindly provided by the Structural Genomic Consortium (https://www.thesgc.org/). To estimate the IC_50_ wild-type and *Bap1*-knockout murine pancreatic cancer cell lines were plated at a density of 2–4 × 10^4^ cells per well in triplicate in 96-well plates and treated with compounds as indicated in the corresponding figures with concentrations ranging from 0.3125 to 20 μM (2-fold dilution series). Three days later, cell viability was assessed with the CellTiter-Glo 2.0 Cell Viability Assay. The plates were read using a GloMax luminometer, and light signal was normalized within (dimethylsulfoxide-treated cells) to adjust for experimental variations. Curve-fitting and IC_50_ values were calculated using Prism5.

### Statistical analysis

Data are presented as mean ± SEM, unless otherwise indicated in the figure legends. Significance was analyzed using two-tailed Student’s *t*-test. A *p*-value < 0.05 was considered statistically significant. To compare more than two experimental groups, one-way analysis of variance was used followed by Tukey’s multiple comparison post-hoc test between groups. A *χ*^2^-test was used to assess the significance of the BAP1 status and clinical features in the analysis of pancreatic cancer TMA. In the Kaplan–Meier plots, the Log-rank (Mantel–Cox) test was used to estimate the median survival and compare the experimental cohorts. For human data, the best expression cut-off for BAP1 was used to stratify patients into “low” and “high” expression groups. Contingency tables were analyzed using two-sided Fisher’s exact test. Statistical analysis took place using GraphPad PRISM version 5.01. For univariate Cox regression analysis, we used the “survival” package version 2.42–3 in R (https://cran.r-project.org/web/packages/survival/index.html) to estimate the HR and 95% CI. In the analysis of the TCGA-PAAD cohort we plotted the “Overall Survival” using raw data from https://portal.gdc.cancer.gov/ or linked portals http://www.cbioportal.org/ and http://xena.ucsc.edu/ (censored event = 0; death event = 1). In the analysis of the TCGA-PAAD cohort, patients TCGA-HZ-7289, TCGA-FB-A7DR, TCGA-HZ-8638, TCGA-3A-A9IR, TCGA-3A-A9IO, TCGA-3A-A9IL, TCGA-IB-7654, TCGA-2L-AAQM, TCGA-3A-A9IJ, TCGA-3A-A9IN, TCGA-3A-A9IS, TCGA-2J-AABP, and TCGA-3A-A9IV were excluded, because the pathology reports indicated that the tumors were of neuroendocrine origin, a distinct malignancy from PDA. We included patients diagnosed with IPMNs, a patient diagnosed with ACC (TCGA-HZ-7918), as well as patient TCGA-FZ-5922 who was diagnosed with pancreatitis and low-grade PanIN, but harbored both *KRAS* and *BAP1* mutations. We queried the cBioPortal using Onco Query Language as follows: “Gene name: GAIN AMP HOMDEL HETLOSS MUT”. For patients lacking information for CNAs and mutations in cBioPortal, we employed the NCI's Genomic Data Commons portal (https://portal.gdc.cancer.gov/) and Xena browser (https://xenabrowser.net/). Finally, interrogation of pathology reports downloaded from http://www.cbioportal.org/ also revealed that several patients (TCGA-FB-AAQ1, TCGA-3A-A9I5, TCGA-H8-A6C1, TCGA-FB-AAPP, TCGA-HZ-A77O, TCGA-FB-A5VM, TCGA-IB-AAUO, TCGA-FB-AAQ6, and TCGA-FZ-5922) with heterozygous loss of BAP1 had histological findings indicative of chronic pancreatitis.

### Reporting summary

Further information on research design is available in the Nature Research Reporting Summary linked to this article.

## Supplementary information

Supplementary Information

Reporting Summary

## Data Availability

Data that support the findings of this study have been deposited in the Gene Expression Omnibus (GEO; https://www.ncbi.nlm.nih.gov/geo/) under the super series accession number GSE120127 (https://www.ncbi.nlm.nih.gov/geo/query/acc.cgi?acc=GSE120127). Raw and processed Affymetrix data from murine and human pancreatic cancer cell lines have been deposited in the Gene Expression Omnibus repository under accession number GSE120075 (https://www.ncbi.nlm.nih.gov/geo/query/acc.cgi?acc=GSE120075), and GSE120078 (https://www.ncbi.nlm.nih.gov/geo/query/acc.cgi?acc=GSE120078), respectively. Raw and processed data from murine ChIP-seq data sets for H3K4me3, H3K27me3, H3K27ac, H3K4me1, H2AK119Ub, and H2BK120Ub have been deposited in the Gene Expression Omnibus repository under accession number GSE121709 (https://www.ncbi.nlm.nih.gov/geo/query/acc.cgi?acc=GSE121709). Raw and processed data from human ChIP-seq data sets for BAP1 and H2AK119Ub have been deposited in the Gene Expression Omnibus repository under accession number GSE120460 (https://www.ncbi.nlm.nih.gov/geo/query/acc.cgi?acc=GSE120460). Raw ChIP-seq data for H3K4me3 (ENCFF346XSZ), POLR2ASer5 (ENCFF065NPZ), H3K36me3 (ENCFF356QYS), and H3K27me3 (ENCFF072NKY) for PANC1 were downloaded from ENCODE (https://www.encodeproject.org/). For data mining, we used The NCI's Genomic Data Commons portal (https://portal.gdc.cancer.gov/), cBioPortal (http://www.cbioportal.org/), the Xena browser (https://xenabrowser.net/), the Cancer Digital slide archive (http://cancer.digitalslidearchive.net/), the International Cancer Genome Consortium (http://icgc.org/), COSMIC (http://cancer.sanger.ac.uk/cosmic), Tabula Muris (https://tabula-muris.ds.czbiohub.org/images/Pancreas-facs-cell_ontology_class-tsne.png), R2: Genomics Analysis and Visualization Platform (http://r2.amc.nl), the Cancer Cell Line Encyclopedia (https://portals.broadinstitute.org/ccle), and CellMinerCDB (https://discover.nci.nih.gov/cellminercdb/). The source data underlying Figs. 1b–e, 2c, e, 3a–c, 4a, 5b–d, h, 6c, f, and 7a–e, and Supplementary Figs. 1b, c, g, h, 2b, d, e, i, 3a, b, d, e, 5a, c–l, and 7a, b, f–i are provided as a Source Data file. All other relevant data are available in the Article, Supplementary Information or from the corresponding author upon reasonable request.

## Source Data

Source Data
