# Supplementary material for: BAP1 is a haploinsufficient tumor suppressor linking chronic pancreatitis to pancreatic cancer in mice
Source: Nat Commun. 2020 Jun 15;11:3018. doi: 10.1038/s41467-020-16589-8 (PMC7295806; doi:10.1038/s41467-020-16589-8)
Supplement: Supplementary file 1 — Supplementary Information [file 41467_2020_16589_MOESM1_ESM.pdf]

BAP1 is a haploinsufficient tumor suppressor linking chronic pancreatitis to pancreatic cancer in mice

Perkail et al.

Figure S1

Perkail et al

a

BAP1 status vs sample purity (TCGA-PAAD)

	High Purity	Low Purity	Total
**Het_Loss**	28	14	42
**Diploid**	47	59	106
Total	75	73	148

Fisher's exact test p = 0.0178

b

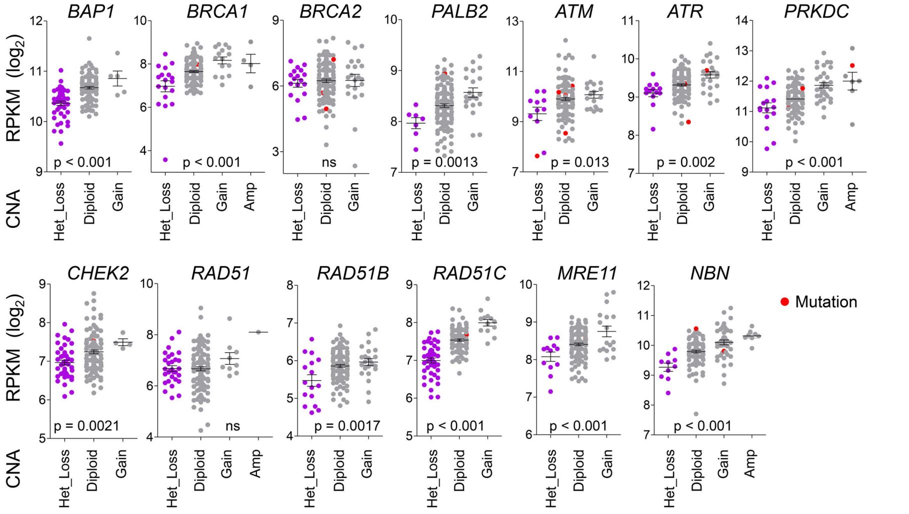

c

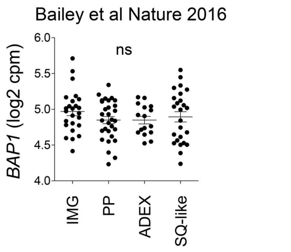

d

BAP1 CNA in pancreatic cancer

Pancreatic Ductal Adenocarcinoma | Acinar Cell Carcinoma | Cystic Neoplasms of the pancreas

Study	Mutation	Het_Loss	Deletion	Cumulative #	
TCGA-PAAD (**Cancer Cell 2017**)	1/155 (0.65%) *	45/155 (29%)	1/155 (0.65%)	46/155 (29.7%)	179/647 **27.7%**
UTSW (Witkiewicz et al. **Nature Communications 2015**)%	0/109 (0%)	39/109 (35.8%)	4/109 (3.7%)	43/109 (39.5%)	
MSK-IMPACT (Zehir et al. **Nature Medicine 2017**)	2/384 (0.5%) $	89/384 (23.17%)	1/384 (0.025%)	90/384 (23.4%)	
Acinar Cell Carcinoma (Jäkel et al. **Nature Communications 2017**) cohort I	1/22 (4.6%)	8/22 (36.4%)	1/22 (4.6%)	10/22 (45.5%)	22/55 **40%**
Acinar Cell Carcinoma (Jäkel et al. **Nature Communications 2017**) cohort II	N/A	6/19 (31.6%)	0/19 (0%)	6/19 (31.6%)	
Acinar Cell Carcinoma (Zehir et al. **Nature Medicine 2017**) MSK-IMPACT	0/14	6/14 (42.8%)	0/14	6/14 (42.8%)	
IPMN & MCN (Zehir et al. **Nature Medicine 2017**) MSK-IMPACT	0/10	2/10 (20%)	0/10	2/10 (20%)	
Solid Pseudopapillary Neoplasms (Amato et al. **Journal of Pathology 2018**) $$	1/5 @	N/A	N/A	1/5 (20%)	
Pancreatic Intraductal Tubulopapillary Neoplasms (Bastruck et al. **Modern Pathology 2017**)	2/22 (9.1%)	1/22 (4.5%)	0/22	3/22 (13.6%)	

Cumulative: number of patients with at least one genetic alteration (mutation, LOH, or deletion)

* One patient (TCGA-FZ-5922-01) simultaneously carries a "Splice_site" mutation and LOH of *BAP1*

$ Both patients simultaneously carry mutations and LOH of *BAP1*

% Use of matched primary and tumor specimens.

$$ Use of matched primary and tumor specimens. IHC also revealed an inverse correlation between metastatic disease and BAP1 staining.

@ Frame shift mutation and generation of an out-of-frame transcript

N/A data not available

e

Acinar Cell Carcinoma (Jäkel et al. Nat. Commun. 2017)

Cohort I

Gene	Locus.ID	Cytoband	10T	11T	12T	13T	14T	15T	16T	17T	18T	19T	1T	20T	21T	22T	23T	3T	4T	5T	6T	7T	8T	9T
BAP1	8314	3p21.1	0	-1	-1	0	0	-1	-1	0	0	-1	0	0	0	0	-1	1	0	-1	0	-2	-1	0
BRCA2	675	13q13.1	0	-1	-1	0	0	-1	0	-1	-1	0	-1	0	-1	0	0	1	-2	0	0	0	1	0
PALB2	79728	16p12.2	0	-1	1	0	0	-1	0	-1	-1	0	-1	-1	-1	0	0	0	0	-1	0	0	-1	0
BRCA1	672	17q21.31	0	-1	0	0	0	-1	0	0	0	0	0	0	0	0	-1	0	-1	-1	0	0	-1	0

Cohort II

Gene	Locus ID	Cytoband	K1 T	K10 T	K11 T	K12 T	K13 T	K14 T	K15 T	K16 T	K18 T	K19 T	K2 T	K20 T	K3 T	K4 T	K5 T	K6 T	K7 T	K8 T	K9 T
BAP1	8314	3p21.1	0	-1	0	0	0	-1	0	0	0	-1	0	-1	0	0	-1	0	0	-1	0
BRCA2	675	13q13.1	-1	-1	0	0	0	-1	0	0	-1	-1	-1	1	0	-1	0	0	-1	1	0
PALB2	79728	16p12.2	0	-1	0	-1	-1	-1	0	0	0	-1	0	1	0	-1	0	-1	0	-1	0
BRCA1	672	17q21.31	0	1	0	-1	0	0	0	0	0	-1	0	0	0	0	0	-1	0	-1	0

Gain | Het_Loss | Hom_Deletion

f

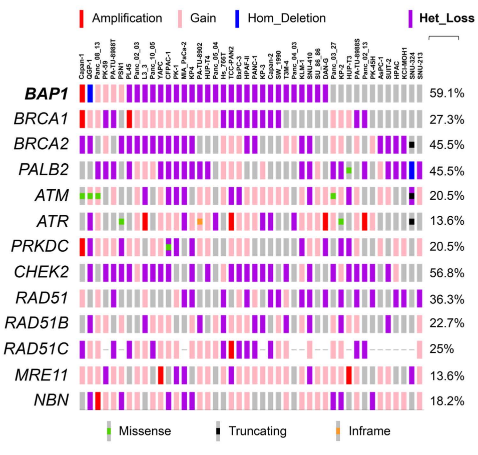

g

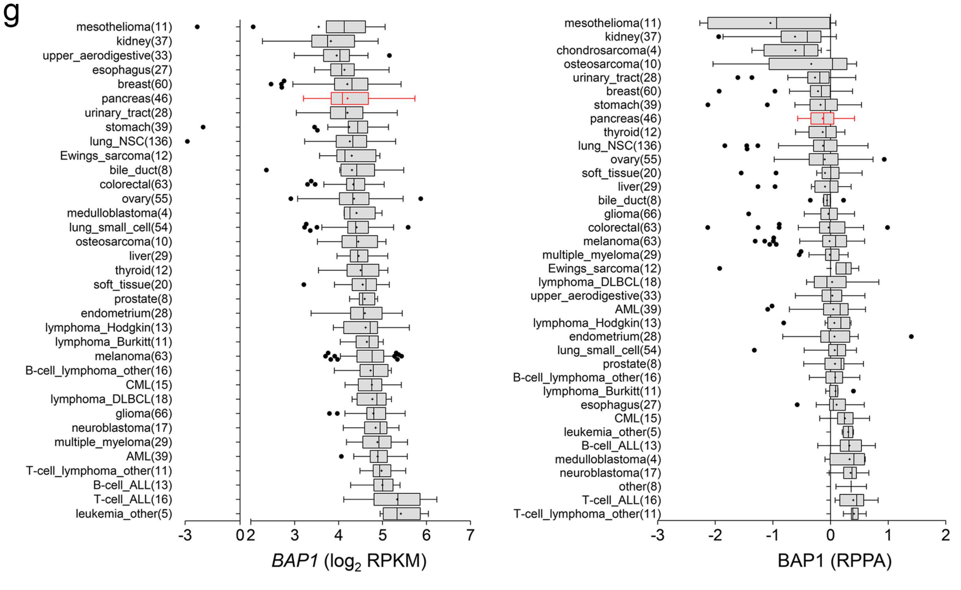

continued on next page

Figure S1_continued

Perkail et al

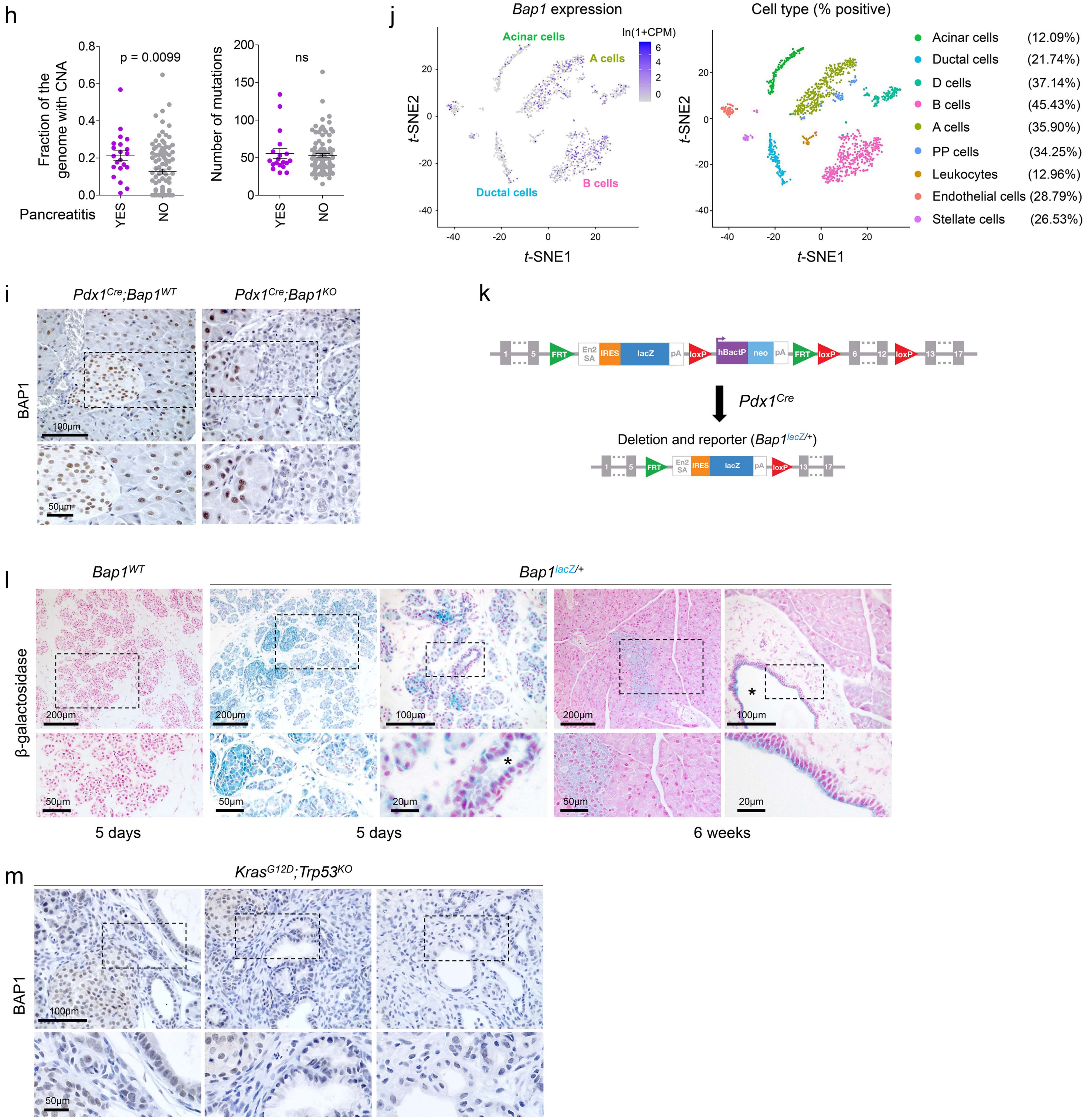

Supplementary Figure 1

a, Contingency table showing the status of *BAP1* in the TCGA-PAAD cohort in relationship to sample purity (high vs low). Significance was determined by a two-sided Fisher's exact test. **b**, Scatter dot plots (mean ± SEM) showing the relationship between the expression and copy number alterations (CNA) of the indicated genes in TCGA-PAAD patients (n=154). Statistical significance was determined by one-way ANOVA. Each dot represents a patient. **c**, Scatter dot plot (mean ± SEM) showing the expression of *BAP1* in different subtypes of pancreatic cancer. Statistical significance was determined by one-way ANOVA, followed by Tukey's multiple comparison post-hoc test between groups. ns, non-significant. IMG, Immunogenic; PP, Pancreatic Progenitor; ADEX, Aberrantly Differentiated Endocrine Exocrine; SQ, Squamous. Each dot represents a patient. **d**, Table showing the frequency of mutations, heterozygous loss, and deletion of *BAP1* in Pancreatic Ductal Adenocarcinoma (PDA, green), Acinar Cell Carcinoma (ACC, orange), and cystic tumor of the pancreas (blue). IPMN, Intraductal Papillary Mucinous Neoplasm; MCN, Mucinous Cystic Neoplasm. **e**, Oncoprint showing patients with copy number alterations of the indicated DNA repair genes in two different cohorts of Acinar Cell Carcinomas. **f**, Oncoprint showing the frequency of copy number alterations and mutations of the indicated DNA repair genes in human pancreatic cancer cell lines from the CCLE. The percentage of cell lines with heterozygous loss for each gene is shown on the right. **g**, Box plots showing *BAP1* expression (left) and protein levels (right) across the compendium of more than 1,000 cell lines from the CCLE stratified based on the tissue of origin. Pancreatic cancer cell lines are highlighted in red. Box limits indicate the first and third quartiles, the band inside the box indicates the median, whiskers extend 1.5 times the interquartile range, the + sign indicates the mean and dots represent the outliers. **h**, Scatter dot plots (mean ± SEM) showing the fraction of genome carrying copy number alterations (CNA, left) and mutations (right) in TCGA-PAAD patients with a history of chronic pancreatitis (yes versus no). Statistical significance was determined by a two-tailed unpaired Student's t test. Each dot represents a patient. **i**, IHC to validate the specificity of the BAP1 antibody (Cell Signaling #13187) using 8-10 week old knockout pancreata. BAP1 is expressed in pancreatic islets and weakly in exocrine pancreas of wild-type animals. The *Pdx1*^{Cre} strain expresses *Cre* recombinase in a stochastic pattern causing a mosaic loss of BAP1 expression which is accompanied by loss of acinar architecture. **j**, t-SNE plots of gene expression derived from 1564 single pancreas cells isolated from three female and four male 12-15 week old mice and colored based on *Bap1* expression (left) and their identity (right). Raw data were obtained from the Tabula Muris consortium (https://tabula-muris.ds.czbiohub.org/). The percentage of cells that show detectable *Bap1* expression is shown in the parentheses. **k**, Schematic of the "knockout-first allele" targeting vector encompassing a *lacZ* reporter flanked by *FRT* sites and *Bap1* exons 6-12 flanked by *loxP* sites. *LacZ* expression is driven by the endogenous promoter. **l**, β-galactosidase staining in pancreata of the indicated genotype and age. Asterisks indicate ducts. **m**, IHC

for BAP1 in pancreatic tumors from 8-10 week old *Pdx1^{Cre};Kras^{G12D};Trp53^{KO}* mice. ns, non-significant. Source data are provided as a Source Data file.

Figure S2

Perkail et al

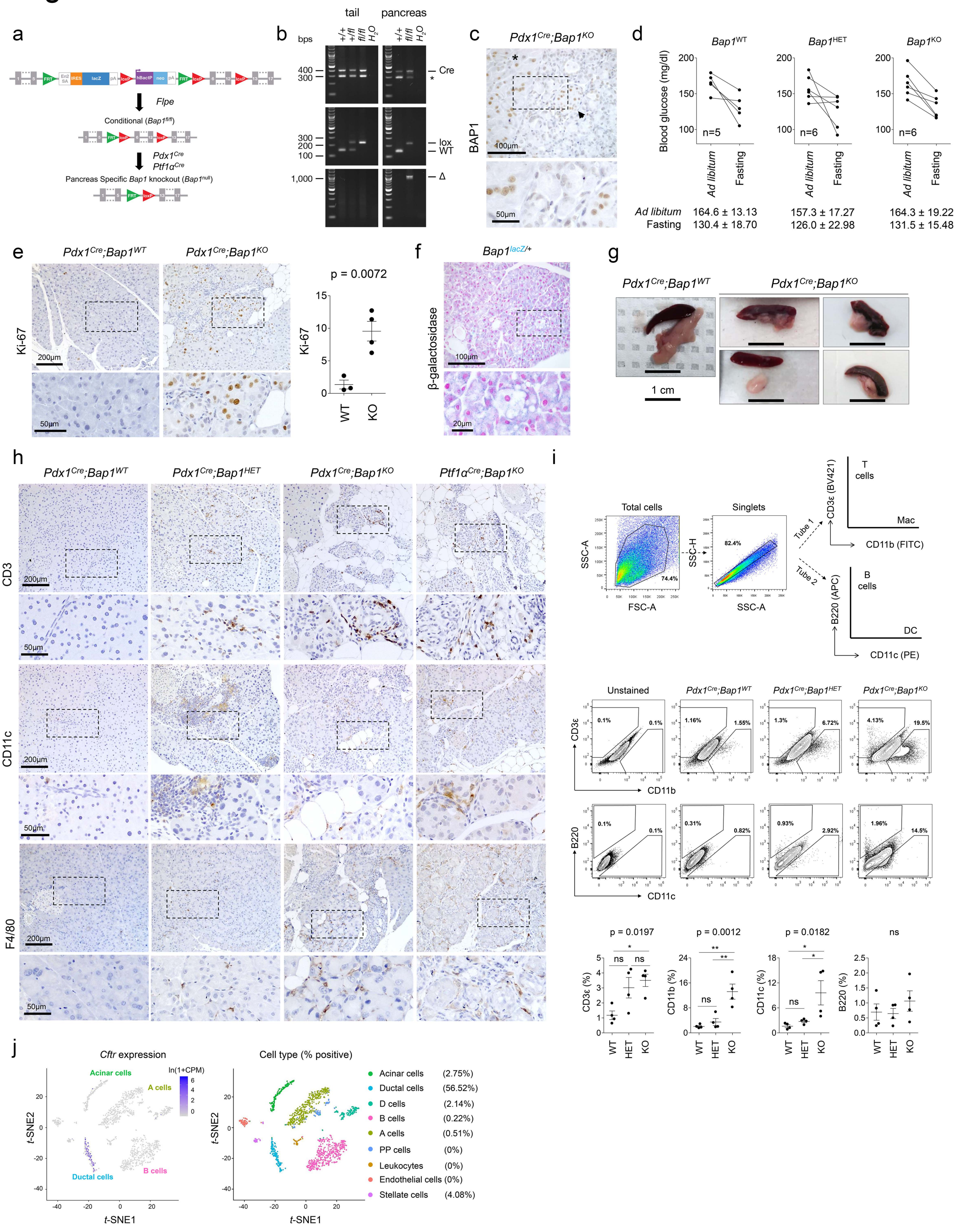

Supplementary Figure 2

a, Schematic of the "knockout-first allele" targeting vector and sequential crosses with *β-Actin*^{FLPe} and then *Pdx1*^{Cre} and *Ptf1α*^{Cre} strains to generate pancreas-specific *Bap1* conditional knockout mice. **b**, Genomic DNA was isolated from tails and pancreata of wild-type, heterozygous, and knockout mice and subjected to PCR with primers specific for the *cre*, *lox*, and *Δ* (recombined) alleles. Recombination was detected in the pancreata of *Pdx1*^{Cre}*;Bap1* ^{floxed} mice. The asterisk indicates the internal control to monitor PCR efficiency. **c**, IHC for Bap1 in 8-10 week old knockout pancreata. Asterisk indicates normal acini and arrow points to an islet. **d**, Blood glucose levels (mg/dl) in 8-20 week old mice of the indicated genotypes. Mice were either fed *ad libitum* or fasted overnight. The glucose levels were within the physiological range and no statistically significant difference was observed among the genotypes. **e**, IHC for Ki-67 in 8-10 week old wild-type (n=3) and *Bap1* knockout (n=4) pancreata. Right: Scatter dot plot showing the number of positive cells (mean ± SEM) per 0.1mm^2 of tissue per mouse. Each dot represents a mouse. Statistical significance was determined by a two-tailed unpaired Student's t test. **f**, β-galactosidase staining of *Bap1*$^{lacZ/+}$ pancreata from a 40 week old mouse. Sections were counterstained in nuclear fast red. The inset shows histological alterations similar to the ones presented in *Pdx1*^{Cre}*;Bap1*^{HET} mice, including alterations in acinar architecture and luminal dilation of acini. **g**, Pictures of pancreata isolated from 25-30 week old wild-type and *Bap1* knockout mice. **h**, IHC for CD3 (T-cells), CD11c (dendritic cells), and F4/80 (macrophages) in 20-30 week old wild-type and *Bap1* deficient pancreata. **i**, Top: Gating strategy to quantify the number of infiltrating immune cells. Middle: Representative flow cytometry analysis of 20-30 week old wild-type and *Bap1* deficient pancreata gated on live singlets showing the relative frequency of T cells (Cd3ε$^+$), dendritic cells (Cd11c$^+$), macrophages (Cd11b$^+$), and B cells (B220$^+$). Bottom: Scatter dot plots showing the percentage of the indicated immune cell types (mean ± SEM) in *Bap1* deficient pancreata (n=4 mice for each genotype). Each dot represents a mouse. Statistical significance was determined by one-way ANOVA with p values shown on the top of each plot, followed by Tukey's multiple comparison post-hoc test between groups. ns, non-significant; *, $p < 0.05$; **, $p < 0.01$. **j**, t-SNE plots of gene expression derived from 1564 single pancreas cells isolated from three female and four male 12-15 week old mice and colored based on *Cftr* expression (left) and their identity (right). Raw data were obtained from the Tabula Muris consortium (https://tabula-muris.ds.czbiohub.org/). The percentage of cells that show detectable *Cftr* expression is shown in the parentheses. Source data are provided as a Source Data file.

Figure S3

Perkail et al

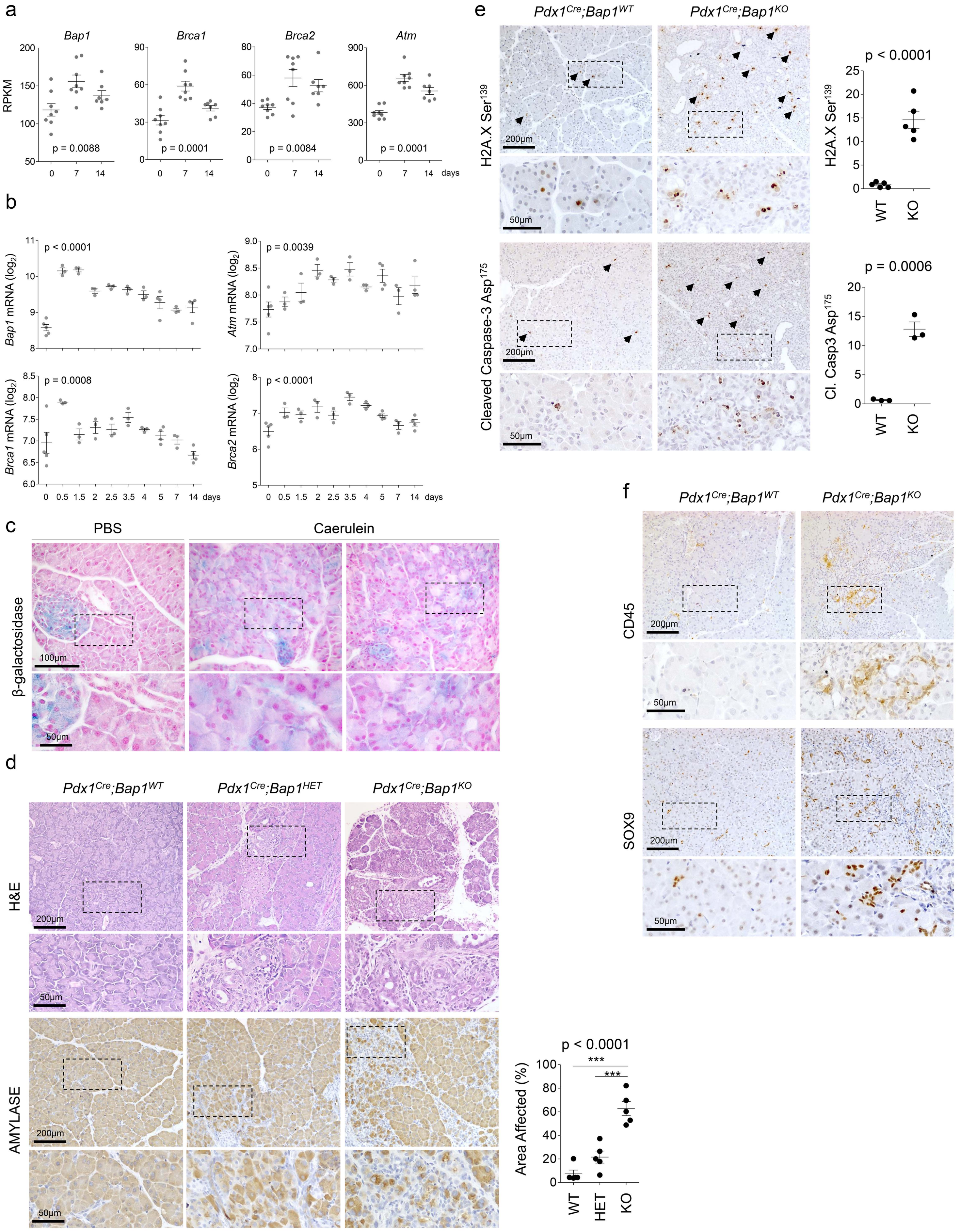

Supplementary Figure 3

a-**b**, Scatter dot plots (mean ± SEM) showing the expression of the indicated genes in pancreata of caerulein treated mice. Raw data were obtained from (**a**) GSE99774 (n=8, n=8, and n=7 mice for days 0, 7, and 14, respectively) and (**b**) GSE65146 (n=5 mice for days 0, 5, 14 and n=3 mice for days 0.5 to 4 and 7). Statistical significance was determined by one-way ANOVA. **c**, β-galactosidase staining in 15 week old *Bap1*$^{lacZ/+}$ pancreata five days post caerulein treatment. **d**, Six week old mice of the indicated genotypes were treated with caerulein to induce pancreatitis. Pancreas regeneration was assessed by H&E and Amylase staining five days later. The scatter dot plot (mean ± SEM) shows the percentage of area with persistent pancreatitis (n=5 mice for WT, HET, and KO). Statistical significance was determined by one-way ANOVA with p values shown on the top of each plot, followed by Tukey's multiple comparison post-hoc test between groups. ***, $p < 0.001$. **e**-**f**, Six week old wild-type and *Bap1* knockout mice were treated with caerulein to induce pancreatitis. Five days later pancreata were analyzed by IHC for the indicated proteins. Scatter dot plots in (**e**) show the number of positive cells (mean ± SEM) per 0.1 mm^2 of tissue per mouse (H2A.XSer^{139}: WT n=5 and KO n=5 mice; Cl. Caspase Asp^{175}: WT n=3 and KO n=3 mice). Each dot represents a mouse. Statistical significance was determined by a two-tailed unpaired Student’s t test. Source data are provided as a Source Data file.

Figure S4

Perkail et al

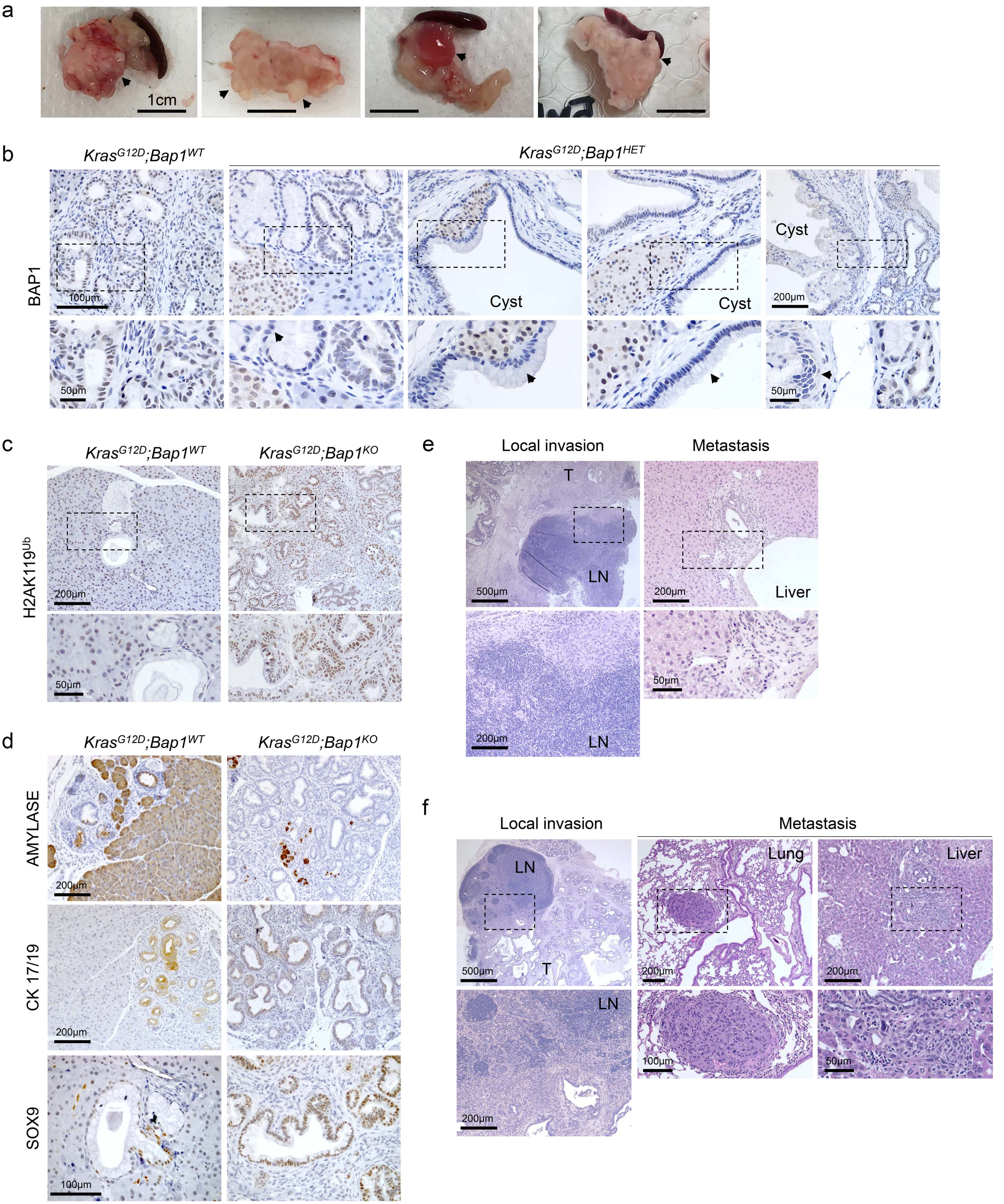

continued on next page

Figure S4_continued

Perkail et al

g

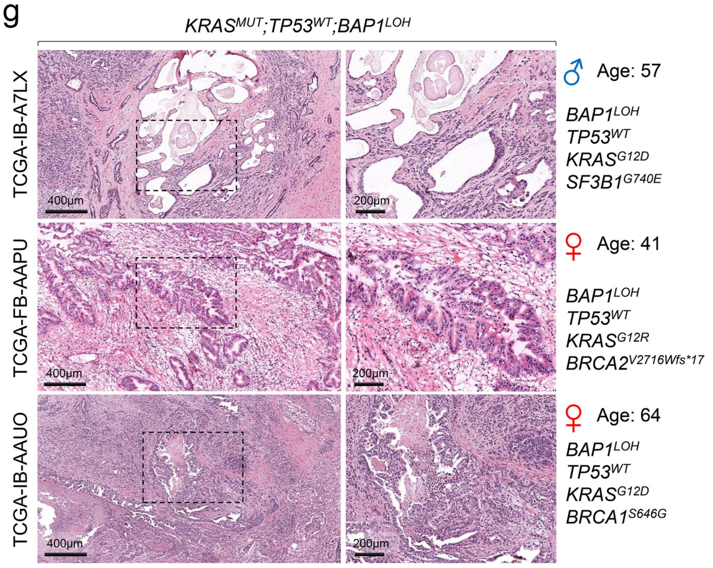

h

Relationship between *BAP1* CNA and other genetic alterations in the TCGA-PAAD cohort

A	B	Log2 Odds Ratio	p-Value	q-Value	Tendency
BAP1_HETLOSS	**GNAS: MUT**	**<-3**	**0.031**	0.246	**Mutual exclusivity**
BAP1_HETLOSS	ATM: MUT	-0.685	0.561	0.734	Mutual exclusivity
BAP1_HETLOSS	RNF43: MUT	-0.5	0.504	0.711	Mutual exclusivity
BAP1_HETLOSS	SMAD4	-0.036	0.551	0.734	Mutual exclusivity
BAP1_HETLOSS	KRAS: MUT	-0.015	0.602	0.734	Mutual exclusivity
BAP1_HETLOSS	SF3B1: MUT	0.343	0.636	0.734	Co-occurrence
BAP1_HETLOSS	CDKN2A	0.576	0.181	0.474	Co-occurrence
BAP1_HETLOSS	TP53	0.596	0.216	0.494	Co-occurrence
BAP1_HETLOSS	BRCA1: MUT	1.357	0.488	0.711	Co-occurrence
BAP1_HETLOSS	BRCA2: MUT	1.357	0.488	0.711	Co-occurrence

i

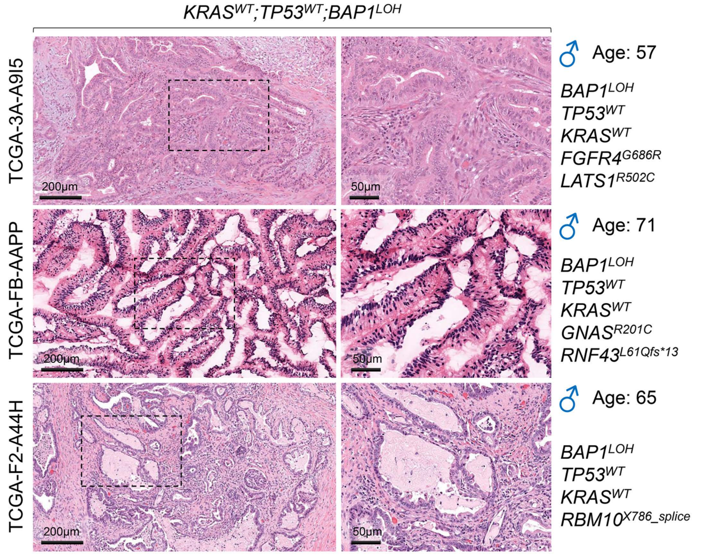

l

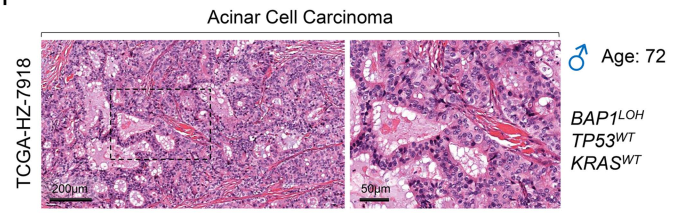

j

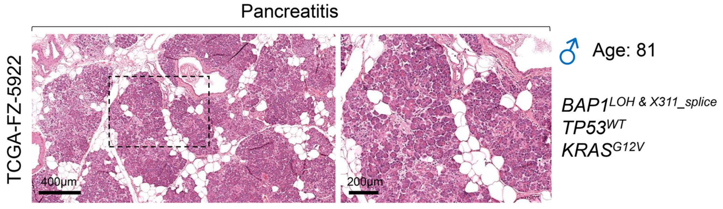

k

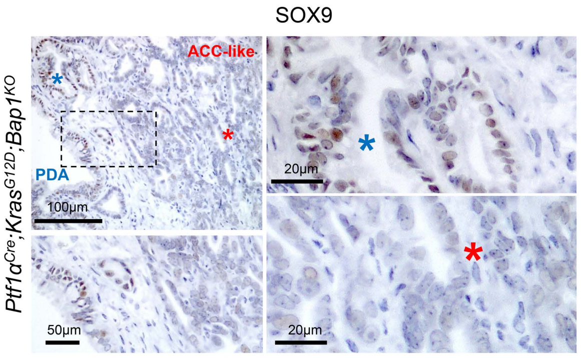

Supplementary Figure 4

a, Pictures of *Pdx1*^{Cre}*;Kras*^{G12D}*;Bap1*^{KO} pancreata at the time of necropsy (10-15 week old). Arrows point to fluid-filled cysts. **b**, IHC for BAP1 in 15-20 week old wild-type and heterozygous mice. Arrows point to the absence of BAP1 staining in cystic lesions. **c**-**d**, IHC for the indicated proteins in 6-8 week old wild-type and *Bap1* knockout *Pdx1*^{Cre}*;Kras*^{G12D} pancreata. **e**-**f**, H&E staining showing examples of local invasion and metastases in 10-15 week old *Kras*^{G12D}*;Bap1*^{KO} (**e**) and 30-35 week old *Kras*^{G12D}*;Bap1*^{HET} mice (**f**). LN, lymph node; T, tumor. **g**, Representative H&E staining of human pancreatic cancer specimens carrying mutant *KRAS* and *BAP1* LOH with intact *TP53*. **h**, Table referring to Fig. 2d showing the co-occurrence between heterozygous loss of *BAP1* with mutations and copy number alterations of oncogenic drivers and tumor suppressor in pancreatic cancer. Statistical significance was determined by one-sided Fisher Exact test and q values were derived from Benjamini-Hochberg FDR correction test. **i**, Representative H&E staining of specimens carrying *BAP1* LOH with intact *KRAS* and *TP53*. **j**, H&E staining of the only specimen carrying mutant *BAP1* and LOH. **k**, IHC for SOX9 in 25-30 week old *Ptf1α*^{Cre}*;Kras*^{G12D}*;Bap1*^{KO} pancreata showing glandular ACC-like lesions stain negative for SOX9 (red asterisk), whereas adjacent PDA stains positive (blue asterisk). **l**, H&E staining of the only human specimen diagnosed as acinar cell carcinoma in the TCGA-PAAD cohort. In (**g**) (**i-j**) and (**l**) H&E pictures were retrieved from the TCGA-PAAD Cancer Digital Slide Archive (http://cancer.digitalslidearchive.net/) representing MCN (TCGA-IB-A7LX and TCGA-F2-A44H), IPMN (TCGA-FB-AAPU, TCGA-IB-AAUO, TCGA-3A-A9I5, and TCGA-FB-AAPP), pancreatitis (TCGA-FZ-5922), and Acinar Cell Carcinoma (TCGA-HZ-7918). LOH, loss of heterozygosity.

Figure S5

Perkail et al

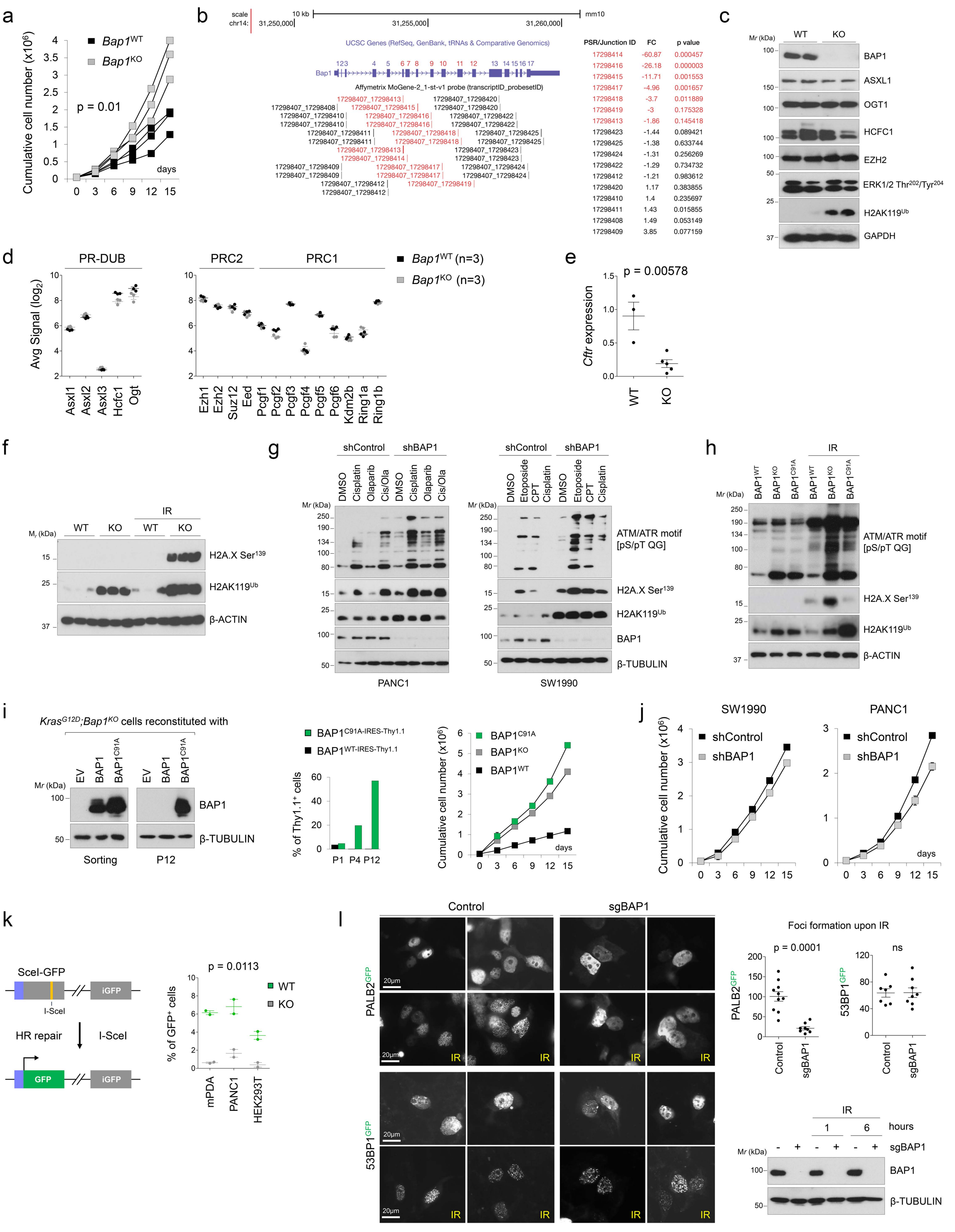

Supplementary Figure 5

a, Pancreas cell lines were independently established from 10-15 week old wild-type (n=3) and *Bap1* knockout (n=3) *Pdx1^{Cre};Kras^{G12D}* mice. 5 x 10^4 cells were plated in triplicate for each cell line and counted every three days. The line graph shows the cumulative cell number (mean ± SD). Statistical significance was determined by a two-tailed unpaired Student's t test**. b**, Affymetrix exon level analysis of the murine *Bap1* locus in cell lines established from knockout mice from the *Pdx1^{Cre};Kras^{G12D}* cohort. The position of the Affymetrix probes is shown at the bottom. Probes in red highlight the floxed exons (6-12). The table shows the p value and fold-change between knockout and wild-type cell lines for each probe spanning the *Bap1* locus. **c**, Western blots showing the levels of the indicated proteins in wild-type and *Bap1* knockout cell lines established from the *Pdx1^{Cre};Kras^{G12D}* cohort. **d**, Dot plot showing the log_2 transformed expression of the indicated transcripts (mean ± SEM) in wild-type (n=3) and *Bap1* knockout cell lines (n=3) established from the *Pdx1^{Cre};Kras^{G12D}* cohort and analyzed by Affymetrix microarrays. **e**, Dot plot showing the relative expression (mean ± SEM) of *Cftr* in wild-type (n=3) and *Bap1* knockout (n=5) cell lines independently established from the *Pdx1^{Cre};Kras^{G12D}* cohort and assessed in triplicates by qRT-PCR. Statistical significance was determined by a two-tailed unpaired Student's t test**. f**, Three independent wild-type and *Bap1* knockout cell lines established from the *Pdx1^{Cre};Kras^{G12D}* cohort were exposed to 10 Gy of IR. Two days later cell lysates were collected and analyzed for the indicated proteins by western blotting. **g**, PANC1 and SW1990 cells expressing short hairpins against *BAP1* or control were treated with the indicated compounds (Cisplatin 10 μM, Olaparib 20 μM, Etoposide 10 μM, or Camptothecin (CPT) 2 μM) for 6 hours. Whole cell lysates were analyzed by western blotting. **h**, *Pdx1^{Cre};Kras^{G12D};Bap1^{KO}* cells reconstituted with wild-type or catalytically inactive mutant of BAP1 (BAP1^{C91A}) were exposed to 10 Gy of IR. One hour later whole cell lysates were collected and analyzed by western blotting. **i**, *Pdx1^{Cre};Kras^{G12D};Bap1^{KO}* cells were infected with retroviruses expressing wild-type or mutant BAP1^{C91A} along with Thy1.1 (CD90.1) through an IRES element. Left: Thy1.1^{+} cells were analyzed by western blotting for the level of BAP1 following sorting and after passaging. Middle: Infected cells were plated at low densities and analyzed by flow cytometry at the indicated passages for the presence of Thy1.1 marker. The bar graph shows the percentage of Thy1.1^{+} cells. Expression of mutant BAP1^{C91A} drives positive selection. Right: 1 x 10^5 sorted Thy1.1^{+} cells expressing wild-type or mutant BAP1^{C91A} were plated in duplicate and counted every three days. The line graph shows the cumulative

cell number (mean ± SD). **j**, 5 x 10^4 cells of the indicated cell lines treated with shBAP1 were plated in triplicate and counted every three days. The line graph shows the cumulative cell number (mean ± SD). **k**, Left: schematic of HR repair assay. The reporter consists of two inactive GFP genes in tandem. SceI-GFP contains a stop codon in the I-SceI endonuclease site, and iGFP is an N-terminally truncated GFP fragment. Cleavage of SceI-GFP by I-SceI endonuclease introduces a double-stranded break which can be repaired by HR using the downstream iGFP sequence as a template, restoring GFP expression. Right: Dot plot shows the percentage of GFP positive cells (mean ± SEM) for two independent experiments performed for the indicated cell lines (mPDA; murine pancreatic cell line). The detected GFP correlates with the efficiency of HR. Statistical significance was determined by a two-tailed unpaired Student's t test. The p value was calculated comparing all WT (n=6) vs all KO (n=6) cell lines. **l**, Left: Control and sgBAP1 treated HEK293T cells were transfected with constructs expressing GFP-tagged PALB2 and 53BP1. Live cells were analyzed for the subcellular localization of the GFP-tagged proteins before and six hours after exposure to 10 Gy of IR. Right: Scatter dot plot on top shows the number of foci (mean ± SEM) upon IR. Each dot represents a nucleus expressing GFP-tagged PALB2 or 53BP1. Statistical significance was determined by a two-tailed unpaired Student's t-test. Western blot shows BAP1 protein levels in the same cell lines before after exposure to IR. Source data are provided as a Source Data file.

Figure S6

Perkail et al

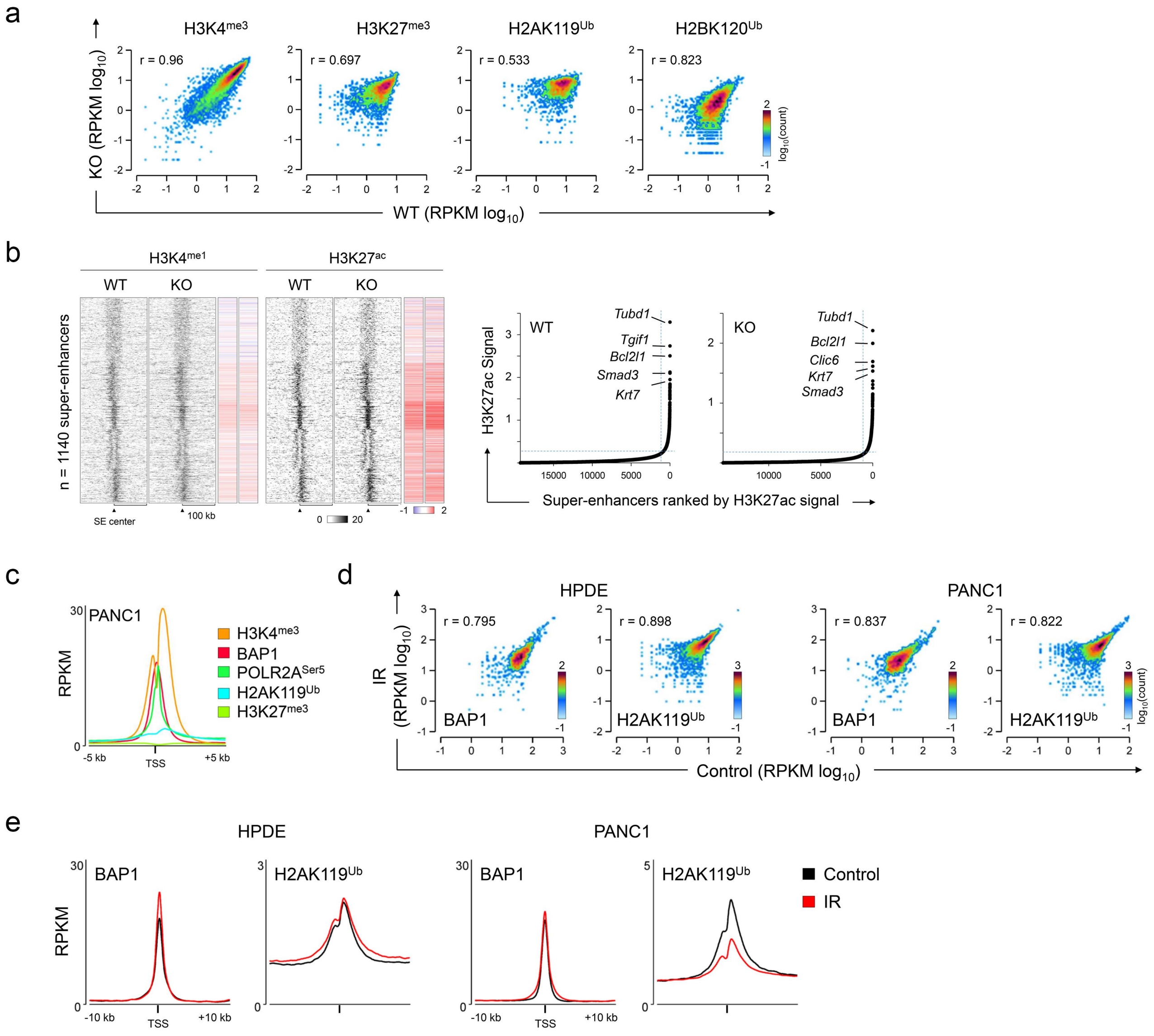

Supplementary Figure 6

a, 2D scatter plots showing the normalized signal intensity (RPKM, reads per kilobase per million mapped reads) of the indicated histone modifications in *Pdx1^{Cre};Kras^{G12D}* wild-type and *Bap1* knockout cells. The color bar shows the count density. r, Pearson's correlation coefficient. **b**, Left: Composite heatmap showing the distribution and signal intensity of H3K4me1 and H3K27ac over super-enhancers identified in *Pdx1^{Cre};Kras^{G12D}* wild-type and *Bap1* knockout cells. *K*-means clustering (*K* = 5) was performed based on the H3K27ac signal that defines active super-enhancers. Each horizontal line represents the normalized signal intensity over a SE. A ± 100 kb window is shown for each SE. The color heat maps show the normalized signal intensity. The grayscale bar shows the normalized RPKM. Right: Rank ordering of super-enhancers based on the normalized H3K27ac signal intensity. The genes linked to the top super-enhancers are highlighted in each cell line. **c**, Read density profiles of the indicated histone marks and BAP1 over the TSS ± 5 kb in PANC1 cells. The y axis shows the mean RPKM. **d**, 2D scatter plots showing the normalized RPKM for BAP1 and H2AK119Ub over the TSS genome-wide in the indicated cell lines before and one hour after exposure to 10 Gy of IR. The color bar shows the count density. r, Pearson's correlation coefficient. **e**, Read density profiles of H2AK119Ub and BAP1 over the TSS ± 10 kb in the in the indicated cell lines before and one hour after exposure to 10 Gy of IR. The y axis shows the mean RPKM.

Figure S7

Perkail et al

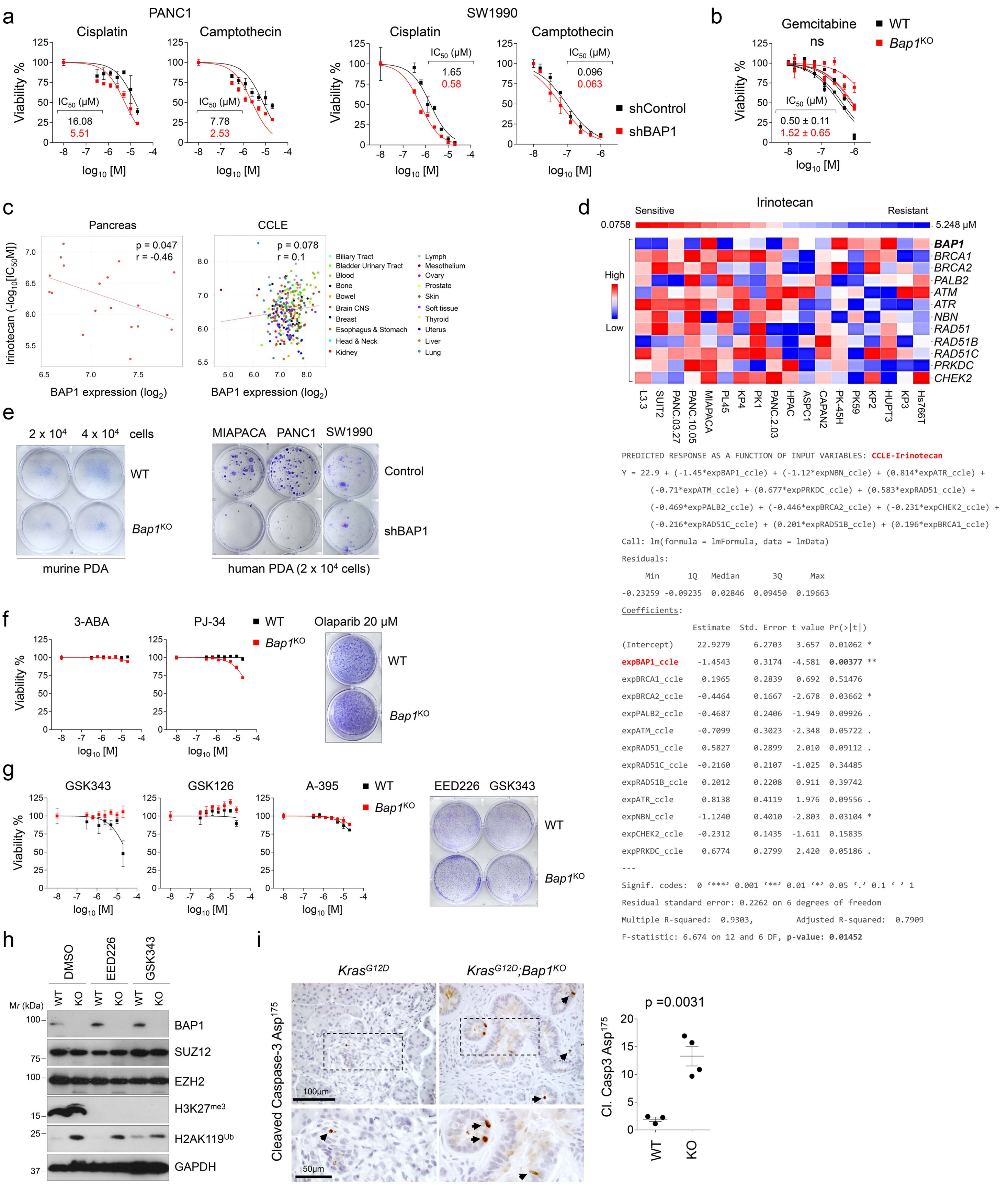

Supplementary Figure 7

a, Estimation of IC_{50} values for cisplatin and camptothecin in PANC1 (left) and SW1990 (right) treated with shBAP1. The graphs show cell viability assessed in triplicates (mean ± SEM) for the indicated concentrations of compounds. **b**, Estimation of IC_{50} values for gemcitabine in wild-type and *Bap1* knockout pancreatic cell lines established from the $Pdx1^{Cre};Kras^{G12D}$ cohort. The average IC_{50} values (mean ± SEM) are shown from three independent cell lines. Statistical significance was determined by a two-tailed unpaired Student's t test. ns, non-significant. **c**, Pearson's rank correlation of Irinotecan sensitivity in relationship to BAP1 expression in human pancreatic (left) and non-pancreatic cancer (right) cell lines from the CCLE. Raw data were obtained from the CellMinerCDB (https://discover.nci.nih.gov/cellminercdb/). **d**, Top: Heat map indicating Irinotecan drug sensitivity of human pancreatic cancer cell lines (CCLE) in relation to the expression of DNA repair genes. Cell lines were ranked from sensitive (left) to resistant (right). The color bar on the left indicates the relative gene expression. Bottom: Statistical summary of a multivariate linear regression model of Irinotecan response in relation to the expression of genes involved in DNA repair. Raw data were obtained from the CellMinerCDB (https://discover.nci.nih.gov/cellminercdb/). **e**, Murine (left) and human (right) pancreatic cancer cell lines depleted for BAP1 were plated in six-well plates and exposed to 10 Gy of IR. Two weeks later surviving cells were stained with crystal violet. **f**, Left: $Pdx1^{Cre};Kras^{G12D}$ wild-type and *Bap1* knockout cell lines were treated with increasing doses of the indicated PARP inhibitors, and three days later cells were assessed for viability. Right: $2x10^4$ cells were plated in a six-well plate and treated with 20 μM of Olaparib. Two weeks later surviving cells were stained with crystal violet. **g**, Left: $Pdx1^{Cre};Kras^{G12D}$ wild-type and *Bap1* knockout cell lines were treated for three days with the indicated doses of EZH2 (GSK343 and GSK126) and EED (A-395 and EED226) inhibitors and assessed for viability. Right: $2x10^4$ cells were plated in a six-well plate and treated with 10 μM of the indicated inhibitors. Two weeks later surviving cells were stained with crystal violet. The graphs in (**f**) and (**g**) show the cell viability assessed in triplicates (mean ± SEM) for the indicated concentrations of compounds. **h**, $Pdx1^{Cre};Kras^{G12D}$ wild-type and *Bap1* knockout cell lines were treated for three days with 10 μM of the indicated inhibitors of PRC2. Western blotting confirmed depletion of H3K27me3 but not changes in the levels of EZH2, SUZ12, and H2AK119Ub. **i**, 10-15 week old mice of the indicated genotype (WT n=3 and KO n=4 mice) were exposed to 10 Gy of IR. Three days later mice were euthanized, and pancreata were stained for cleaved Caspase-3. The scatter dot plot shows the number of positive cells (mean ± SEM) per 0.1 mm^2 of tissue per mouse. Each dot represents a mouse. Black arrows point to positively stained cells. Statistical significance was determined by a two-tailed unpaired Student's t test. Source data are provided as a Source Data file.

Supplementary Table 1

List of primers

Genotyping Primers	Sequence (5' -> 3')
**Bap1 loxP F**	CAC CCT GCG TCT GAG AGA AC
**Bap1 loxP R**	AGG TCG GGC TGA AAG ATC AC
**Neo F**	AGG ATC TCC TGT CAT CTC ACC TTG CTC CTG
**Neo R**	AAG AAC TCG TCA AGA AGG CGA TAG AAG GCG
**lacZ F**	ATC ACG ACG CGC TGT ATC
**lacZ R**	ACA TCG GGC AAA TAA TAT CG
**Bap1 Δ F**	ATC TGG GGC CCA CGC TGA GCC GAA TGA AGG
**Bap1 Δ R**	GCT AGG GGT GGG TGA AGG TTG CGA GTG TGT
**KrasG12D F**	CTA GCC ACC ATG GCT TGA GT
**KrasG12D R**	TCC GAA TTC AGT GAC TAC AGA TG
**Cre F**	CCT GGA AAA TGC TTC TGT CCG
**Cre R**	CAG GGT GTT ATA AGC AAT CCC
**Gabra F**	CAA TGG TAG GCT CAC TCT GGG AGA TGA T
**Gabra R**	AAC ACA CAC TGG CAG GAC TGG CTA GG
**p53 loxP F**	GGT TAA ACC CAG CTT GAC CA
**p53 loxP R**	GGA GGC AGA GAC AGT TGG AG
**mouse qPCR Primers**	**Sequence (5' -> 3')**
**mCftr F**	ATG CAG AAG TCG CCT TTG GA
**mCftr R**	GCG TGG ATA AGC TGG GGA TT
**mActB F**	GCT CTA GAC TTC GAG CAG GAG A
**mActB R**	GGC ATA GAG GTC TTT ACG GAT G

Supplementary Table 2

List of primary and secondary antibodies

Primary Antibodies	Company	Catalog number	Application	Dilution (IHC/WB)	Dilution (ChIP)
Amylase	Cell Signaling	3796	IHC	1:2000	N/A
ASXL1	Santa Cruz	sc-85283	WB	1:1000	N/A
CD45	eBioscience	13-0451-82	IHC	1:1000	N/A
CFTR	Proteintech	20738-1-AP	IHC	1:2500	N/A
Cleaved-Caspase 3 Asp175	Cell Signaling	9661	IHC	1:2000	N/A
Cytokeratin 17/19	Cell Signaling	3984	IHC	1:5000	N/A
GAPDH	Cell Signaling	2118	WB	1:2000	N/A
H2A.XSer139	Cell Signaling	9718	IHC/WB	1:1000	N/A
H2AK119Ub	Cell Signaling	8240	IHC/WB/ChIP	1:5000	1:1000
H2BK120Ub	Cell Signaling	5546	ChIP	N/A	1:1000
H3K27me3	Cell Signaling	9733	WB/ChIP	1:2000	1:1000
H3K27ac	Cell Signaling	8173	ChIP	N/A	1:1000
H3K4me1	Cell Signaling	5326	ChIP	N/A	1:1000
H3K4me3	Cell Signaling	9751	ChIP	N/A	1:1000
HCFC1	Cell Signaling	69690	WB	1:1000	N/A
Ki67	Cell Signaling	12202	IHC	1:2000	N/A
MUC1	ThermoFisher	MA5-11202	IHC	1:1000	N/A
MUC2	ThermoFisher	MA5-12345	IHC	1:1000	N/A
MUC5AC	ThermoFisher	MA5-12178	IHC	1:1000	N/A
OGT	Cell Signaling	24083	WB	1:1000	N/A
SOX9	Millipore	ABE2868	IHC/WB	1:5000	N/A
β-ACTIN	Cell Signaling	4970	WB	1:1000	N/A
β-TUBULIN	Cell Signaling	2128	WB	1:1000	N/A
BAP1	Santa Cruz	sc-28383	WB (Human BAP1)	1:2000	N/A
BAP1 (D1W9B)	Cell Signaling	78105	ChIP	N/A	1:300
BAP1 (D7W7O)	Cell Signaling	13271	IHC/(Mouse BAP1)	1:5000	N/A
BAP1 (D1W9B)	Cell Signaling	13187	WB/ChIP	1:1000	1:300
EZH2	Cell Signaling	5246	WB	1:1000	N/A
SUZ12	Cell Signaling	3737	WB	1:1000	N/A
BRCA1	Cell Signaling	9010S	WB	1:1000	N/A
BRCA2	Cell Signaling	10741S	WB	1:1000	N/A
53BP1	Cell Signaling	4937S	WB	1:1000	N/A
anti-FLAG	Biolegend	637319	WB	1:5000	N/A
Phospho-ATM/ATR Substrate Motif [(pS/pT)	Cell Signaling	6966	WB	1:1000	N/A
ERK 1/2 Thr202/Tyr204	Cell Signaling	4370	WB	1:1000	N/A
TRYPSIN-2	Proteintech	15005-1-AP	IHC	1:2000	N/A
CD3-BV-421	Biolegend	100335 (clone 145-2C11)	FC	1:100	N/A
CD11b-FITC	Biolegend	101205 (clone M1/70)	FC	1:100	N/A
CD11c-PE	Biolegend	117307 (clone N418)	FC	1:100	N/A
B220-APC	Biolegend	103211 (clone RA3-6B2)	FC	1:100	N/A
Thy1.1-PE	Biolegend	202523 (clone: OX-7)	FC	1:200	N/A
CD3	Rockland Antibodies	900-C01-B39	IHC	1:2000	N/A
CD11c	Cell Signaling	97585	IHC	1:2000	N/A
**Seconday Antibodies**	**Company**	**Catalog number**	**Application**	**Dilution**	
anti-Armenian Hampster_biotin conjugated	eBioscience	AB_466651	IHC	1:500	
anti-mouse_biotin conjugated	Thermo Fisher	31800	IHC	1:500	
anti-rabbit_biotin conjugated	Thermo Fisher	656140	IHC	1:500	
anit-rat_biotin conjugated	Thermo Fisher	31830	IHC	1:500	
anti-Rabbit_HRP	Thermo Fisher	656120	WB	1:5000	
anti-mouse_HRP	Thermo Scientific	A28177	WB	1:5000	
